# No Casual Relationship Between T2DM and the Risk of Infectious Diseases: A Two-Sample Mendelian Randomization Study

**DOI:** 10.3389/fgene.2021.720874

**Published:** 2021-08-30

**Authors:** Huachen Wang, Zheng Guo, Yulu Zheng, Chunyan Yu, Haifeng Hou, Bing Chen

**Affiliations:** ^1^Intensive Care Unit, The Second Hospital of Tianjin Medical University, Tianjin, China; ^2^Centre for Precision Health, School of Medical and Health Sciences, Edith Cowan University, Joondalup, WA, Australia; ^3^Medical Imaging Department, Longgang District Central Hospital of Shenzhen, Shenzhen, China; ^4^Shandong First Medical University & Shandong Academy of Medical Sciences, Taian, China

**Keywords:** T2DM, sepsis, infections, single-nucleotide polymorphisms, instrumental variable, Mendelian randomization study

## Abstract

**Background:**

In epidemiological studies, it has been proven that the occurrence of type 2 diabetes mellitus (T2DM) is related to an increased risk of infectious diseases. However, it is still unclear whether the relationship is casual.

**Methods:**

We employed a two-sample Mendelian randomization (MR) to clarify the causal effect of T2DM on high-frequency infectious diseases: sepsis, skin and soft tissue infections (SSTIs), urinary tract infections (UTIs), pneumonia, and genito-urinary infection (GUI) in pregnancy. And then, we analyzed the genome-wide association study (GWAS) meta-analysis of European-descent individuals and conducted T2DM-related single-nucleotide polymorphisms (SNPs) as instrumental variables (IVs) that were associated with genome-wide significance (*p* < 5 × 10^–8^). MR estimates were obtained using the inverse variance-weighted (IVW), the MR-Egger regression, the simple mode (SM), weighted median, and weighted mode.

**Results:**

The UK Biobank (UKB) cohort (*n* > 500,000) provided data for GWASs on infectious diseases. MR analysis showed little evidence of a causal relationship of T2DM with five mentioned infections’ (sepsis, SSTI, UTI, pneumonia, and GUI in pregnancy) susceptibility [odds ratio (OR) = 0.99999, *p* = 0.916; OR = 0.99986, *p* = 0.233; OR = 0.99973, *p* = 0.224; OR = 0.99997, *p* = 0.686; OR, 1.00002, *p* = 0.766]. Sensitivity analysis showed similar results, indicating the robustness of causality. There were no heterogeneity and pleiotropic bias.

**Conclusion:**

T2DM would not be causally associated with high-frequency infectious diseases (including sepsis, SSTI, UTI, pneumonia, and GUI in pregnancy).

## Introduction

Infections are responsible for more than 20% of deaths worldwide, with approximately 245,000 sepsis cases in the United Kingdom in 2017 (Rudd et al., 2020). Infections share risk factors with a non-communicable disease or are triggers for them, making it difficult to disentangle their underlying causes (Parks et al., 2012; Remais et al., 2013; Leung et al., 2017).

One such non-communicable disease is type 2 diabetes mellitus (T2DM), a condition affecting close to 465 million people across the globe (Boulton, 2020). In the United Kingdom, the prevalence of diabetes has doubled over the last three decades (Pierce et al., 2009; McGuire et al., 2016). While undoubtedly associated with sepsis and other infections, it is not aligned in all studies (Valdez et al., 1999; Bertoni et al., 2001; Shah and Hux, 2003; Thomsen et al., 2004, 2005; Gregg et al., 2014). However, these findings were subject to confounding and reverse causation bias. Confounding commonly occurs in observational epidemiology when the exposure and the outcome are influenced by a third variable, rather than in their causal pathway. Reverse causation occurs when the outcome influences the exposure. Therefore, traditional epidemiological studies are ill-suited to explore whether there are relationships between T2DM and infections even with statistical adjustments (Lawlor et al., 2004).

Mendelian randomization (MR) is becoming an analytic method that applies genetic proxies as instrumental variables (IVs) and finds stronger evidence for the causal effect of outcomes through an intermediate trait effectively avoiding confounding and reverse causality (Sekula et al., 2016). Given that diabetes or high glucose levels can be prevented by lifestyle changes or medications, if there is a causal relationship between infections and T2DM, this may be a potential strategy for the prevention of infections in T2DM patients. Therefore, we performed a two-sample MR analysis based on statistics from genome-wide association study (GWAS) data to assess whether T2DM was causally associated with the risk of infections (Dimou and Tsilidis, 2018).

## Materials and Methods

### Data Sources and Single-Nucleotide Polymorphism Identification

#### Genetic Association Datasets for Type 2 Diabetes Mellitus Susceptibility

In order to analyze the results, we used a recent meta-analysis of GWAS for T2DM, which compared 62,892 T2DM patients and 596,424 controls of European ancestry with a total of 16 million gene variants, from three contributing studies, including the full cohort release of the UK Biobank (UKB), Diabetes Genetics Replication and Meta-analysis (DIAGRAM), and Genetic Epidemiology Research on Aging (GERA) (Xue et al., 2018). This study identified 139 common variants, and four rare variants are related to T2DM. The role of rare variants in the occurrence of common diseases remains controversial (Gibson, 2012). The latest study showed that the contribution of rare variants to T2DM heritability may be limited (Fuchsberger et al., 2016). Therefore, there are only 139 common and independent variants (*p* < 5 × 10^–8^) included in our analysis. During automatic analysis, two single-nucleotide polymorphisms (SNPs) (rs13234269 and rs11591741) of T2DM were removed for being palindromic with intermediate allele frequencies. As a result, we analyzed 137 SNPs as the IVs. There was no evidence of gender or age heterogeneity in the UKB. To test whether there was a weak bias of the IV, i.e., genetic variation selected as an IV was weakly correlated with exposure, we calculated the *F* statistic [*F* = R^2^(n−k−1)/k(1−R^2^], where R^2^ is the exposure variance explained by the chosen IV; n, sample size; and k, number of IVs). If the *F* statistic is much larger than 10 for an instrument–exposure association, the likelihood of a weak instrument variable bias was small (Staiger and Stock, 1997).

#### Genetic Association Datasets for Infections

Five high-frequency infections related to T2DM were included: sepsis, skin and soft tissue infections (SSTIs), urinary tract infections (UTIs), pneumonia, and genito-urinary infection (GUI) in pregnancy (Miller et al., 2013; Butler-Laporte et al., 2020). To identify whether genetic variants are associated with common infectious diseases, we employed the UKB cohort with genome-wide genotyped data. Then, we derived these summarized data from GWAS analysis in the latest version of infections genetics program in the UKB. To find out genetic variants associated with infections, we used the UKB cohort, which consists of more than 500,000 volunteer participants in the United Kingdom with genome-wide genotyped data.

### Mendelian Randomization Analysis

**FIGURE 1:**
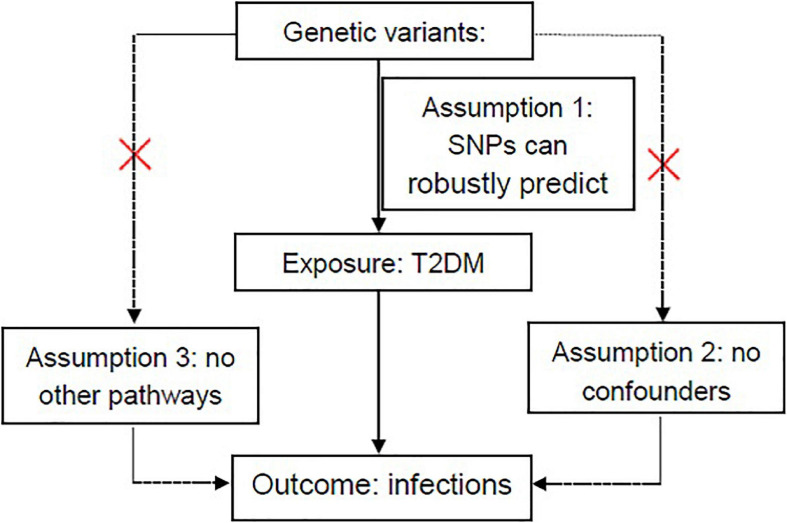
Overview of hypotheses in MR design. Three different assumptions are represented by three paths. Assumption 1: SNPs robustly predict T2DM. Assumption 2: No potential confounders affect infections (sepsis, skin and soft tissue infections, urinary tract infections, pneumonia, and genito-urinary infection in pregnancy). Assumption 3: SNPs impact the abovementioned infections only through T2DM. MR, Mendelian randomization; SNPs, single-nucleotide polymorphisms; T2DM, type 2 diabetes mellitus. Red cross mark in assumption 2 means that genetic variants are not related to known or unknown confounder factors and in assumption 3 it means that genetic variants would have an influence on the outcome (infections) only through exposure (T2DM), not through other pathways.

Because of the lack of comprehensive data in a single cohort (Wensley et al., 2011), we performed a two-sample MR analysis using the two-sample MR software package in R (version 4.0.3) (Burgess et al., 2013; Yavorska and Burgess, 2017) to determine the causal associations between T2DM and common infections. Three important hypotheses need to be demonstrated to ensure that the IVs in MR analysis are valid (Burgess et al., 2015): (1) the SNPs used as IVs can robustly predict T2DM; (2) the SNPs are not associated with potential confounders affecting T2DM and five common infections; and (3) the SNPs influence infections only through their effects on T2DM, but not through any other causal pathways (Figure 1). In this two-sample MR, we employed five methods, including the inverse variance-weighted (IVW), the MR-Egger (MR-Egger) regression, the simple mode (SM), weighted median, and weighted mode (Burgess and Thompson, 2015). Based on the dominance of each MR method, five approaches were conducted to complement each other. In two-sample MR, the IVW method acting as a fixed-effect meta-analysis was usually used. Then, a sensitivity analysis was performed by the weighted mode, the weighted median, the SM, and MR-Egger methods to examine the stability of the causal inference (Burgess et al., 2017; Hartwig et al., 2017). First, the “leave one out” analysis was employed to determine whether causal inference was robustly related to a single SNP. Second, the SNPs were included if they were not significant linkage disequilibrium (LD; *r*^2^ < 0.001) with each other. According to several estimates of different methods, we finally select the results of the principal MR method as the following rules: (1) the results of the IVW method were chosen, when there is no directional pleiotropy shown in the MR estimation (Q statistic, *p*-value > 0.05; MR-Egger intercept, *p*-value > 0.05). (2) The results of the IVW method were chosen, when *p*-value > 0.05 for the *Q* test and directional pleiotropy (MR-Egger intercept, *p*-value < 0.05). (3) The results of the IVW method were chosen, when *p*-value < 0.05 for the *Q* test and directional pleiotropy was detected (MR-Egger intercept, *p*-value < 0.05). An analysis using a web-based application was conducted^1^ (Brion et al., 2013) to calculate the statistical power of the MR. Estimation of detectable odds ratio (OR) was obtained after specifying a power of 80% and a significance of 5%.

## Results

### Instrumental Selection for Type 2 Diabetes Mellitus

One hundred thirty-nine genetic variants were obtained as the IVs according to their GWAS (*p* < 5 × 10^–8^). Finally, we conducted 137 SNPs of T2DM as the IVs after eliminating two SNPs (rs13234269 and rs11591741) for being palindromic with intermediate allele frequencies. Detailed information about 137 SNPs is shown in Supplementary Table 1. F statistic for the instrument–exposure association was 17.17, which is much greater than 10, indicating that the likelihood of a weak instrument variable bias is small.

### Mendelian Randomization Analysis Between Type 2 Diabetes Mellitus and Sepsis Risk

**FIGURE 2:**
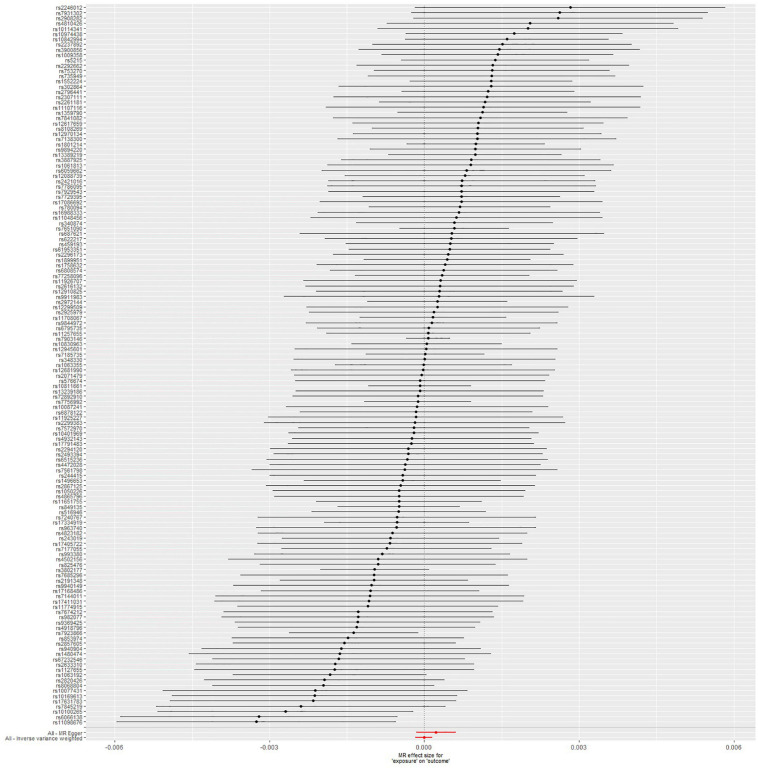
Forest plot: the associations between T2DM-related SNPs and sepsis risk. T2DM, type 2 diabetes mellitus; SNPs, single-nucleotide polymorphisms; OR, odds ratio; CIs, confidence intervals.

The IVW method showed no obvious heterogeneity (*p* = 0.785) between T2DM and sepsis and that there was no association between them (OR = 0.99999; 95% CI, 0.99982–1.00016; *p* = 0.916). The MR-Egger regression analysis provided little evidence to support the association between T2DM and sepsis (OR = 1.00023; 95% CI 0.99985–1.00061; *p* = 0.246). Pleiotropy for T2DM was not observed in the MR-Egger regression analysis (intercept = −1.829 × 10^–5^; *p* = 0.181). Causal association using five different methodological approaches (IVW, MR-Egger, SM, weighted median, and weighted mode) could be found in Table 1. The forest plot showed the OR and a horizontal line representing the sepsis risk of T2DM-related SNPs with 95% CIs (Figure 2). Furthermore, the results of “leave one out” demonstrated that no individual SNPs has any remarkable impact on the overall results by eliminating 137 SNPs one at a time (Supplementary Figure 1). Detailed information about 137 SNPs in sepsis is shown in Supplementary Table 1.

**TABLE 1:** The association between T2DM and infections of odds ratios using different methodological approaches.

Methods	Sepsis		SSTI		UTI		Pneumonia		GUI in pregnancy	
	OR (95% CI)	*P*	OR (95% CI)	*P*	OR (95% CI)	*P*	OR (95% CI)	*P*	OR (95% CI)	*P*
IVW	0.99999 (0.99982, 1.00016)	0.91647	0.99986 (0.99962, 1.00009)	0.23336	0.99973 (0.99929, 1.00017)	0.22371	0.99997 (0.99982, 1.00012)	0.68562	1.00002 (0.99989, 1.00014)	0.76559
MR-Egger	1.00023 (0.99985, 1.00061)	0.24594	1.00021 (0.99967, 1.00076)	0.44507	0.99924 (0.99823, 1.00026)	0.14683	1.00002 (0.99968, 1.00037)	0.88231	0.99990 (0.99963, 1.00018)	0.50136
SM	0.99994 (0.99928, 1.00060)	0.85391	0.99934 (0.99833, 1.00035)	0.20001	0.99975 (0.99819, 1.00138)	0.75606	1.00023 (0.99972, 1.00074)	0.38341	0.99983 (0.99938, 1.00027)	0.44773
Weighted median	1.00006 (0.99974, 1.00039)	0.71720	0.99965 (0.99923, 1.00006)	0.09744	0.99942 (0.99871, 1.00013)	0.11114	0.99996 (0.99968, 1.00024)	0.75989	1.00006 (0.99984, 1.00028)	0.57957
Weighted mode	1.00004 (0.99968, 1.00039)	0.84494	0.99975 (0.99919, 1.00030)	0.37131	0.99922 (0.99833, 1.00011)	0.08985	1.00004 (0.99974, 1.00034)	0.78805	0.99996 (0.99973, 1.00020)	0.75146

*T2DM, type 2 diabetes mellitus; SSTI, skin and soft tissue infections; UTI, urinary tract infection; GUI, genito-urinary infection; IVW, the inverse variance-weighted method; MR-Egger, the Mendelian randomization-Egger method; SM, simple mode; OR, odds ratio; CI, confidence interval; p, p-value; SE, standard error.*

### Mendelian Randomization Analysis Between Type 2 Diabetes Mellitus and Skin and Soft Tissue Infection Risk

**FIGURE 3:**
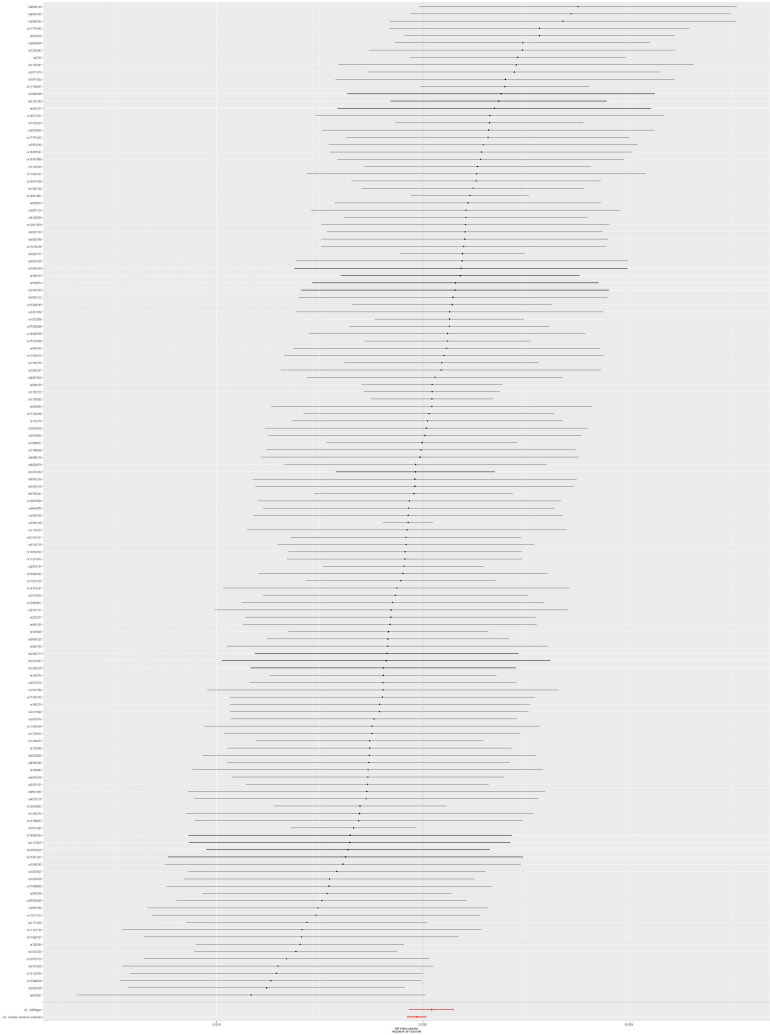
Forest plot: the associations between T2DM-related SNPs and SSTI risk. T2DM, type 2 diabetes mellitus; SSTI, skin and soft tissue infections; SNPs, single-nucleotide polymorphisms; OR, odds ratio; CIs, confidence intervals.

The MR analysis was conducted by using 137 SNPs. We found that there is no causal link between T2DM and SSTI risk according to the results of IVW method (OR = 0.99986; 95% CI, 0.99962–1.00009; *p* = 0.233), without obvious heterogeneity (*p* = 0.678). Moreover, the potential pleiotropy for these 137 SNPs was not observed in the MR-Egger regression analysis (intercept = −2.784 × 10^–5^; *p* = 0.157). Causal association with T2DM and SSTI could be found in Table 1. The forest plot of the SSTI risk is shown in Figure 3. To assess the stability of MR analysis results, we further conducted the sensitivity analysis through a leave-one-out method. The results from sensitivity analysis are shown in Supplementary Figure 2. Detailed information on 137 SNPs in SSTI is shown in Supplementary Table 1.

### Mendelian Randomization Analysis Between Type 2 Diabetes Mellitus and Urinary Tract Infection Risk

**FIGURE 4:**
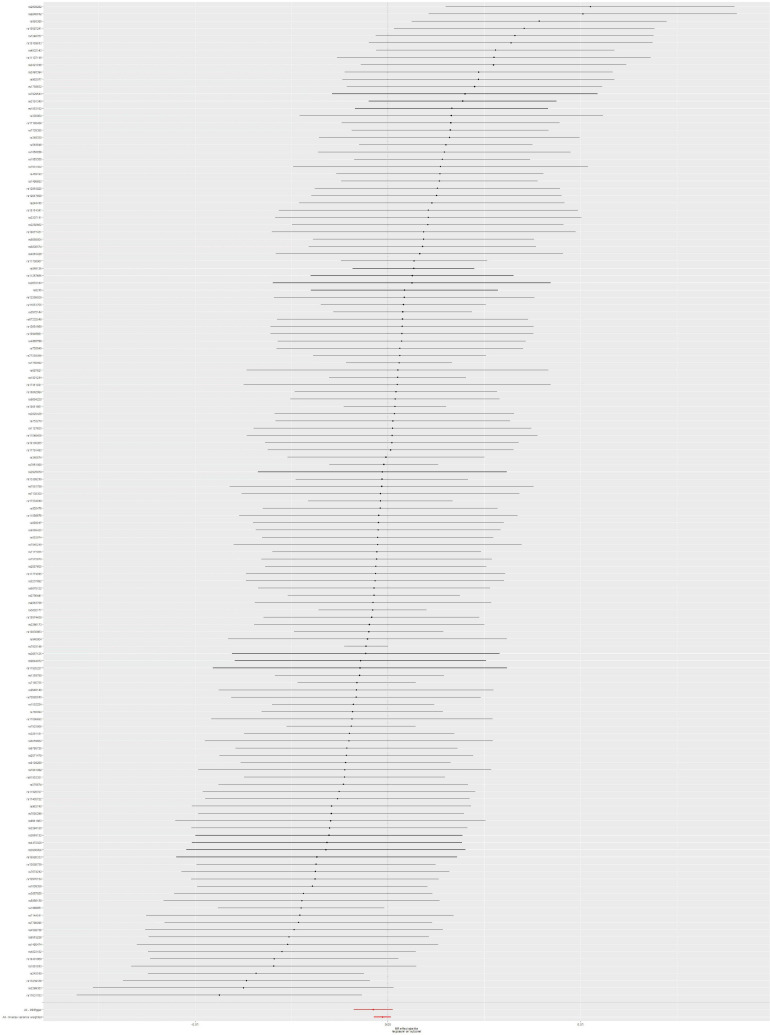
Forest plot: the associations between T2DM-related SNPs and UTI risk. T2DM, type 2 diabetes mellitus; UTI, urinary tract infection; SNPs, single-nucleotide polymorphisms; OR, odds ratio; CIs, confidence intervals.

The results from IVW method were *p*-value = 0.224, OR = 0.99973, and 95% CI = 0.99929–1.00017 (Table 1). According to these results, T2DM was not causally linked to UTI risk. Moreover, there was no observed heterogeneity in this analysis (*p* = 0.557). Also, there is no potential pleiotropy for these SNPs according to the results of the MR-Egger method (intercept = 3.749 × 10^–5^; *p* = 0.303). The forest plot of the UTI risk is shown in Figure 4. To evaluate the robustness of MR results about T2DM and UTI, a sensitivity analysis was also performed for MR studies between T2DM and UTI through the leave-one-out method. The sensitivity analysis results are shown in Supplementary Figure 3, and detailed information about 137 SNPs can be found in Supplementary Table 1.

### Mendelian Randomization Analysis Between Type 2 Diabetes Mellitus and Pneumonia Risk

**FIGURE 5:**
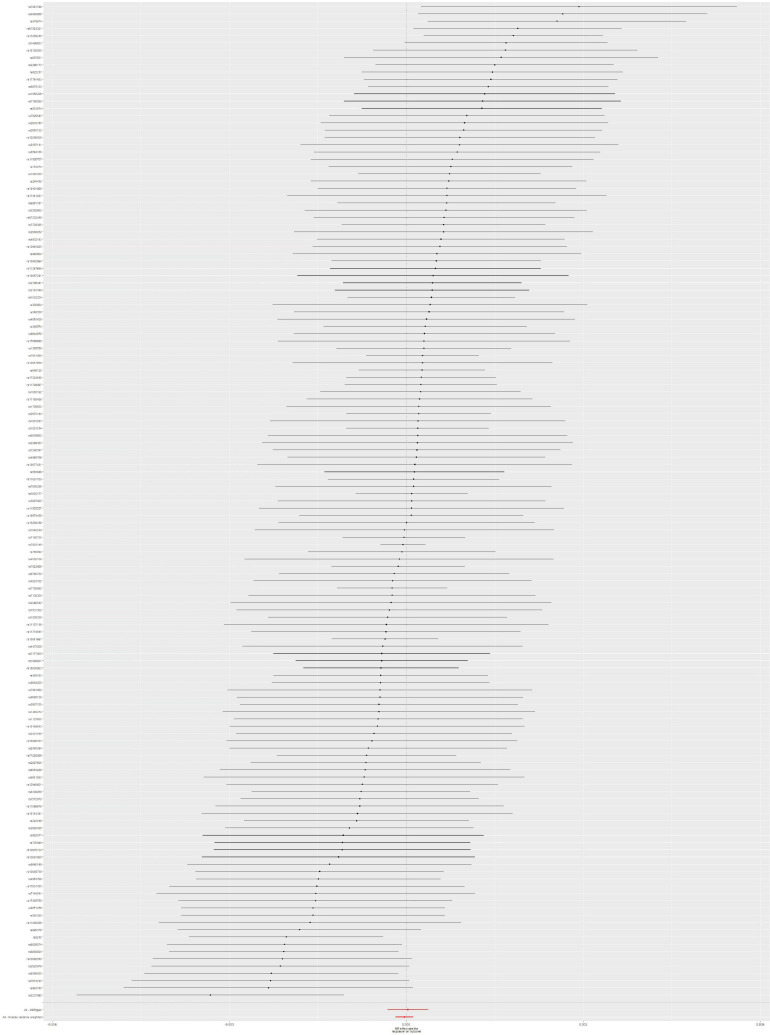
Forest plot: the associations between T2DM-related SNPs and pneumonia risk. T2DM, type 2 diabetes mellitus; SNPs, single-nucleotide polymorphisms; OR, odds ratio; CIs, confidence intervals.

The IVW results (OR = 0.99997; 95% CI, 0.99982–1.00012; *p* = 0.686) indicated that T2DM would not be causally associated with pneumonia risk, without obvious heterogeneity (*p* = 0.667). In addition, the MR-Egger regression (OR = 1.000; 95% CI, 1.000–1.000; *p* = 0.882) and intercept (intercept = −4.416 × 10^–6^; *p* = 0.720) demonstrated that there was no significant pleiotropy of the MR study between T2DM and pneumonia (Table 1). According to the forest plot, there was no causal association between T2DM and pneumonia risk (Figure 5). The sensitive analysis using leave-one-out approach could be obtained in Supplementary Figure 4. Supplementary Table 1 shows SNP-related details.

### Mendelian Randomization Analysis Between Type 2 Diabetes Mellitus and Genito-Urinary Infection Risk in Pregnancy

**FIGURE 6:**
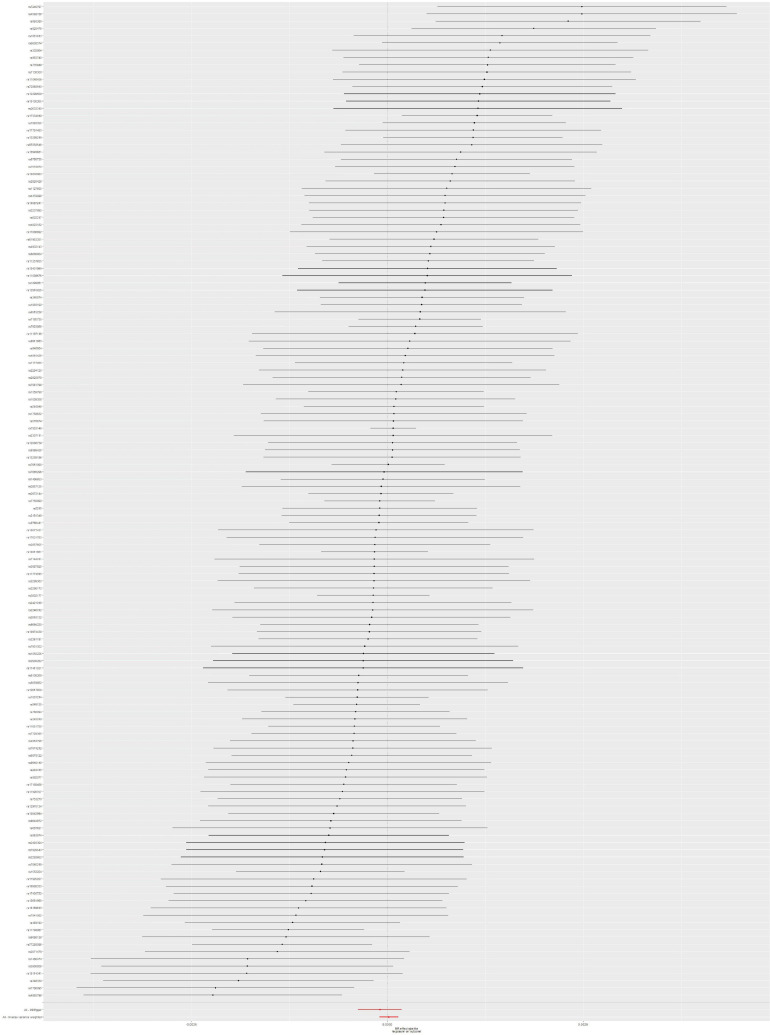
Forest plot: the associations between T2DM-related SNPs and GUI risk in pregnancy. T2DM, type 2 diabetes mellitus; GUI, genito-urinary infection; SNPs, single-nucleotide polymorphisms; OR, odds ratio; CIs, confidence intervals.

We conducted an MR analysis between T2DM and GUI in pregnancy using R package Two Sample MR. IVW result (OR = 1.00002; 95% CI, 0.99989–1.00014; *p* = 0.766) indicated that T2DM was not causally linked to GUI risk in pregnancy, without specifying heterogeneity (*p* = 0.125). According to the MR-Egger result (OR = 0.99990; 95% CI, 0.99963–1.00018; *p* = 0.501 and intercept = 8.848 × 10^–6^; *p* = 0.374), there was no significant pleiotropy in the MR study between T2DM and GUI (Table 1). The forest plot against GUI risk of pregnancy is shown in Figure 6. The sensitive analysis using leave-one-out approach showed that no individual SNPs has any remarkable impact on the overall results by eliminating 137 SNPs one at a time (Supplementary Figure 5). Detailed information about SNPs is obtained in Supplementary Table 1. After >80% power with 5% significance was specified, the estimation of the detectable effect sizes of sepsis, SSTI, UTI, pneumonia, and GUI in pregnancy was 0.99999, 0.99986, 0.99973, 0.99997, and 1.00002, respectively.

## Discussion

Type 2 diabetes mellitus has been related to the increase of incidence and mortality of infectious diseases (Benfield et al., 2007; de Miguel-Yanes et al., 2015; Wang et al., 2019). To clarify this association, we conducted a two-sample MR analysis to investigate whether causal effects existed between T2DM and the risk of related infections. The results of our findings support the notion that T2DM alone might not be responsible for the reported positive association with T2DM and infectious diseases (sepsis, SSTI, UTI, pneumonia, and GUI in pregnancy). These discoveries were reliable for some sensitivity analyses.

Our findings were consistent with those reported in earlier researches (Moss et al., 2000; Gong et al., 2005; Esper et al., 2009; Donath et al., 2019). A prospective study demonstrated that diabetes mellitus was a negative predictor of septic shock complicated with acute respiratory distress syndrome (ARDS) (Moss et al., 2000). The incidence of ARDS in patients with diabetes was significantly lower than that in patients without diabetes (25% vs. 47%; OR, 50.33, 95% CI, 0.12–0.90). These results and our MR analysis seem to suggest that there was little evidence to support the genetic role of T2DM in infection development.

More importantly, patients with T2DM usually have higher blood glucose levels than that in healthy people, which may enable them to better tolerate the effects of acute hyperglycemia when inflammation will be occurring. On the contrary, when non-T2DM patients with acute diseases have acute hyperglycemia, the level of circulating inflammatory cytokines increases significantly and induces more inflammatory responses (Yu et al., 2003), which may increase infection risk.

Another possibility is that the susceptibility variations of T2DM are often related to both higher blood glucose levels and low insulin levels. This may lead to vertical pleiotropy. In this case, genetic variants are related to blood glucose and insulin levels on the same biological pathway from T2DM to common infections. Thus, this condition does not violate the MR assumptions. Furthermore, we conducted the MR-Egger regression, which did not show the probability of pleiotropic effects.

Finally, the influence of T2DM itself on infection risk, if any, may be smaller than we thought. The routine epidemiological analysis might have overestimated the real association, possibly due to the influence of uncontrolled factors involving reverse causation or common risk factors.

Our findings indicated that T2DM may not play a major role in the susceptibility of developing common infections. However, from a public health point of view, controlling these common risk factors is undoubtedly important to prevent these two kinds of diseases, because T2DM and common infections have some established changeable risk factors such as obesity and smoking (Haskins et al., 2014; Calfee et al., 2015; Frydrych et al., 2018).

There are several advantages of our several MR analyses in this article. Firstly, we used the large-scale T2DM GWAS summary dataset and five common infections GWAS summary dataset. MR can reduce the impact on population stratification through a large dataset. In addition, we used the five MR methods in this study, which can increase the robustness of the MR results and prevent reverse causal bias. Several pleiotropic analyses were also conducted, which could reduce the pleiotropic influence on the MR results. We further performed the sensitivity analysis by using the leave-one-out method, which can ensure the stability of the MR results.

Some limitations need to be noticed. First, a potential limitation of our study is that some IVs may overlap across infectious diseases. Theoretically, the exposure and outcome studies used in two-sample MR analysis should not involve overlapping participants. However, in practice, the original GWAS studies mixed some samples. Therefore, we used strong instruments (i.e., F statistic much greater than 10) to minimize the bias caused by overlapping (Pierce and Burgess, 2013). Second, to dissect the causal relationships more clearly, employing the causal effects of glycemic traits (notably fasting glucose and insulin, the homeostatic model assessment for insulin resistance and beta-cell function, and HbA1c) on the infectious disease would be more helpful. Unfortunately, we currently lack suitable data to make this assessment more meticulous. Third, despite our best efforts to resolve confounding effects or potential pleiotropic, the possibility still exists. It is worth noting that some of the variants in our study are associated with insulin-related traits rather than glucose, which may result in horizontal pleiotropy. Our MR results may be distorted by the existence of potential horizontal pleiotropy. Instead of using T2DM-susceptible variants, the use of variants associated with glucose levels, such as glucose transporters, may better address this limitation. Fourth, the genetic variants were not related to tested and undetermined confounders, which may affect both T2DM and infections. However, due to the limitations of the method, unmeasured confounding factors or other causal pathways may still impact our results. Fifth, these findings are based on the population of Europe, so it is difficult to represent the general conclusions of other ethnic and regional populations. Thus, future research needs more regional groups and a larger sample size to verify the observed connections.

## Conclusion

Using T2DM-related SNPs as IVs from GWAS data, this MR study found no strong evidence to support the causal associations between T2DM and five common infection risks in the European population. In addition, randomized controlled trials (RCTs) about the association between T2DM and infections in the long-term outcomes are needed.

## Data Availability Statement

The original contributions presented in the study are included in the article/Supplementary Material, further inquiries can be directed to the corresponding author/s.

## Ethics Statement

The article does not contain the participation of any human beings and animals.

## Author Contributions

YZ and CY performed the acquisition of data, analysis, and interpretation of data. HW and ZG performed the acquisition of data, analysis, interpretation of data, drafted the article, and performed the final approval. HH and BC performed the conception and design of the study, critical revision, and final approval. All authors contributed to the article and approved the submitted version.

## Conflict of Interest

The authors declare that the research was conducted in the absence of any commercial or financial relationships that could be construed as a potential conflict of interest.

## Publisher’s Note

All claims expressed in this article are solely those of the authors and do not necessarily represent those of their affiliated organizations, or those of the publisher, the editors and the reviewers. Any product that may be evaluated in this article, or claim that may be made by its manufacturer, is not guaranteed or endorsed by the publisher.

## Footnotes

1
https://shiny.cnsgenomics.com/mRnd/

## Supplementary Material

Supplementary Figure 1 MR leave-one-out sensitivity analysis for “T2DM” on “sepsis”. MR, Mendelian randomization; T2DM, type 2 diabetes mellitus.

Supplementary Figure 2 MR leave-one-out sensitivity analysis for “T2DM” on “SSTI”. MR, Mendelian randomization; T2DM, type 2 diabetes mellitus; SSTI, skin and soft tissue infections.

Supplementary Figure 3 MR leave-one-out sensitivity analysis for “T2DM” on “UTI”. MR, Mendelian randomization; T2DM, type 2 diabetes mellitus; UTI, urinary tract infections.

Supplementary Figure 4 MR leave-one-out sensitivity analysis for “T2DM” on “pneumonia”. MR, Mendelian randomization; T2DM, type 2 diabetes mellitus.

Supplementary Figure 5 MR leave-one-out sensitivity analysis for “T2DM” on “GUI in pregnancy”. MR, Mendelian randomization; T2DM, type 2 diabetes mellitus; GUI, genito-urinary infection.

Supplementary Table 1 Characteristics of 137 genetic variants in T2DM and infections datasets. T2DM, type 2 diabetes mellitus; SNP, single-nucleotide polymorphism; Chr, chromosome; EA, effect allele; NEA, non-effect allele; SE, standard error; SSTI, skin and soft tissue infection; UTI, urinary tract infection; GUI, genito-urinary infection.
