# Supplementary figures and images for: No Casual Relationship Between T2DM and the Risk of Infectious Diseases: A Two-Sample Mendelian Randomization Study
Source: Front Genet. 2021 Aug 30;12:720874. doi: 10.3389/fgene.2021.720874 (PMC8435717; doi:10.3389/fgene.2021.720874)

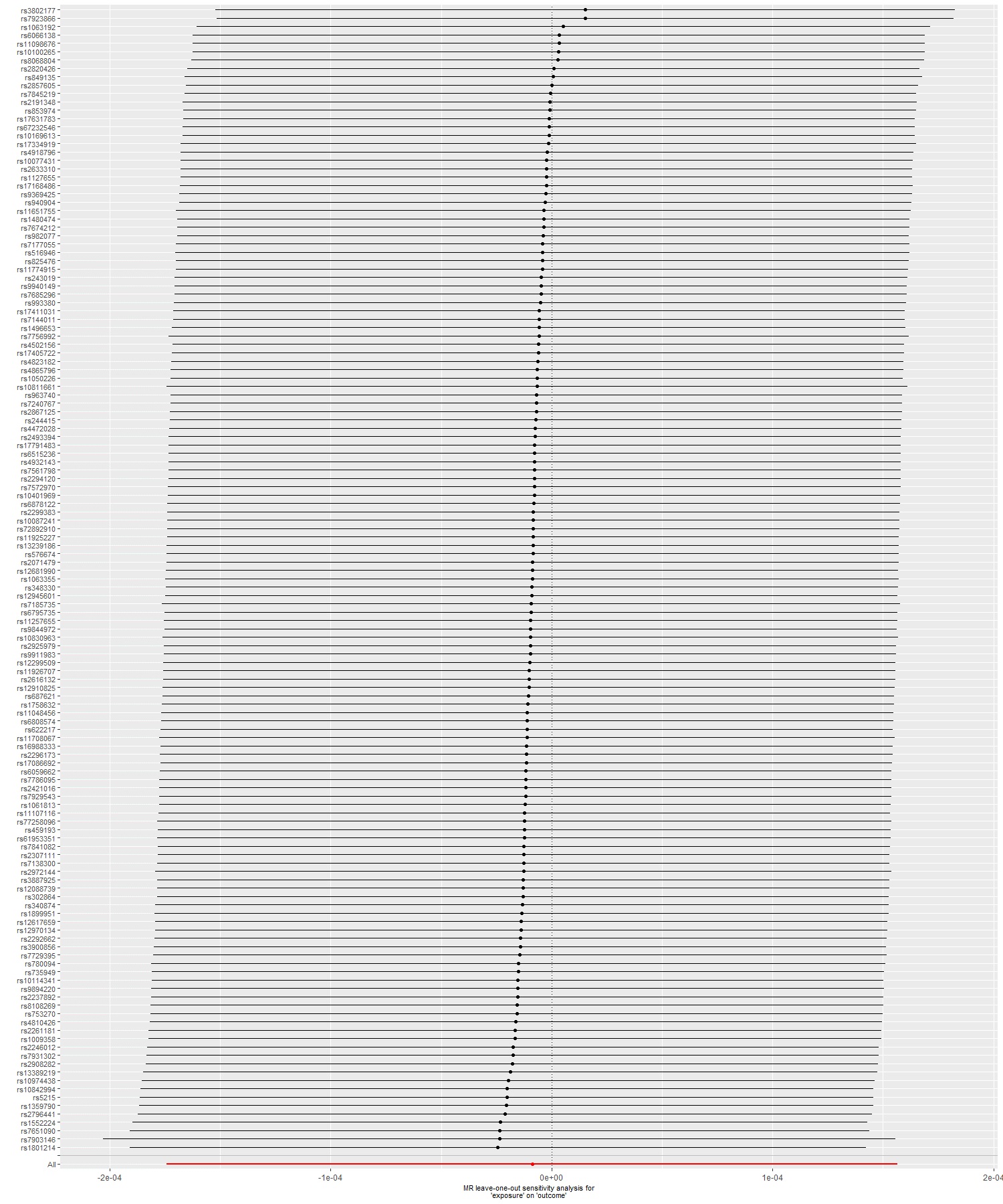

Supplement: Supplementary Figure 1 — MR leave-one-out sensitivity analysis for “T2DM” on “sepsis”. MR, Mendelian randomization; T2DM, type 2 diabetes mellitus. [file Image_1.JPEG]

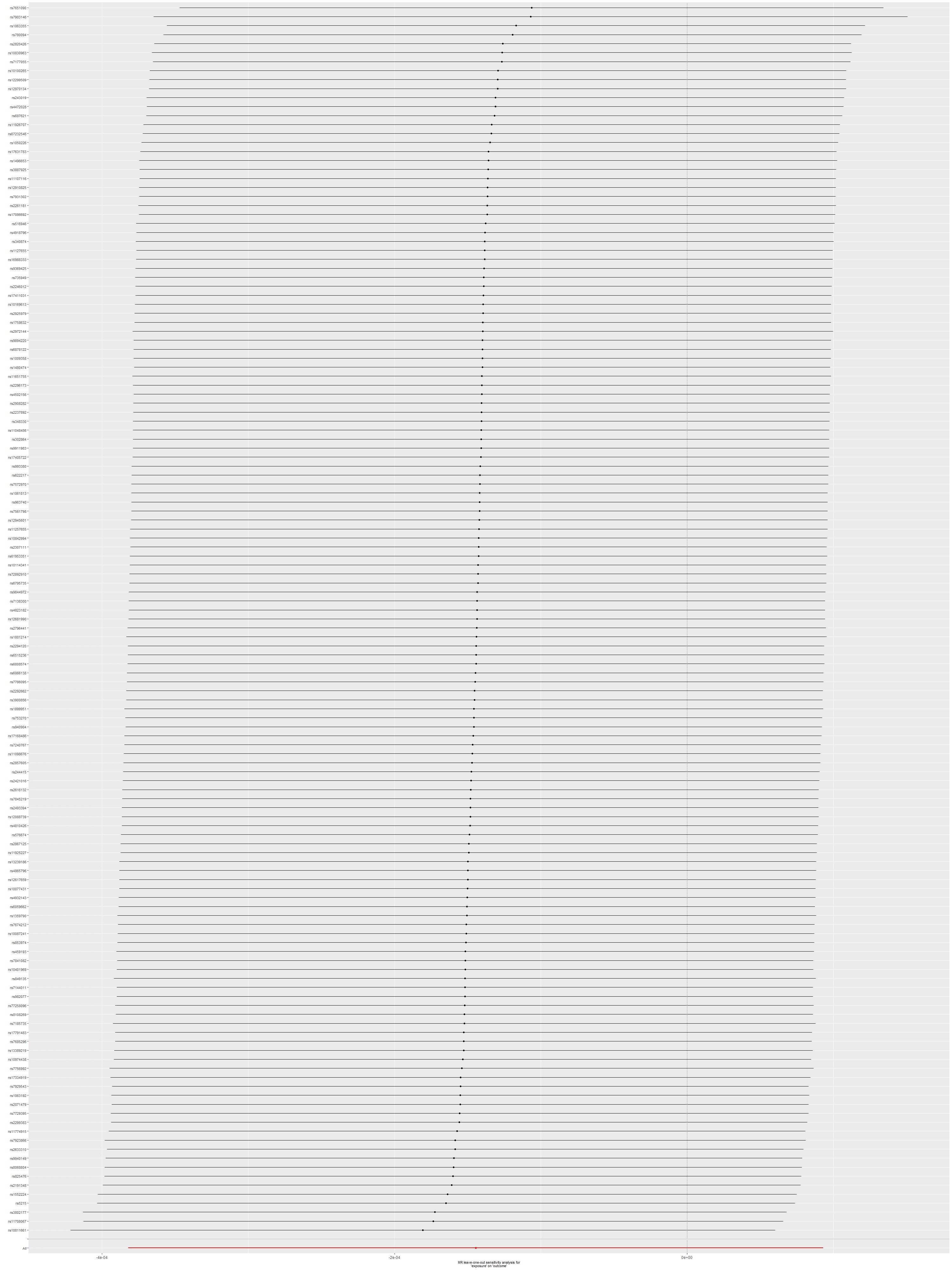

Supplement: Supplementary Figure 2 — MR leave-one-out sensitivity analysis for “T2DM” on “SSTI”. MR, Mendelian randomization; T2DM, type 2 diabetes mellitus; SSTI, skin and soft tissue infections. [file Image_2.JPEG]

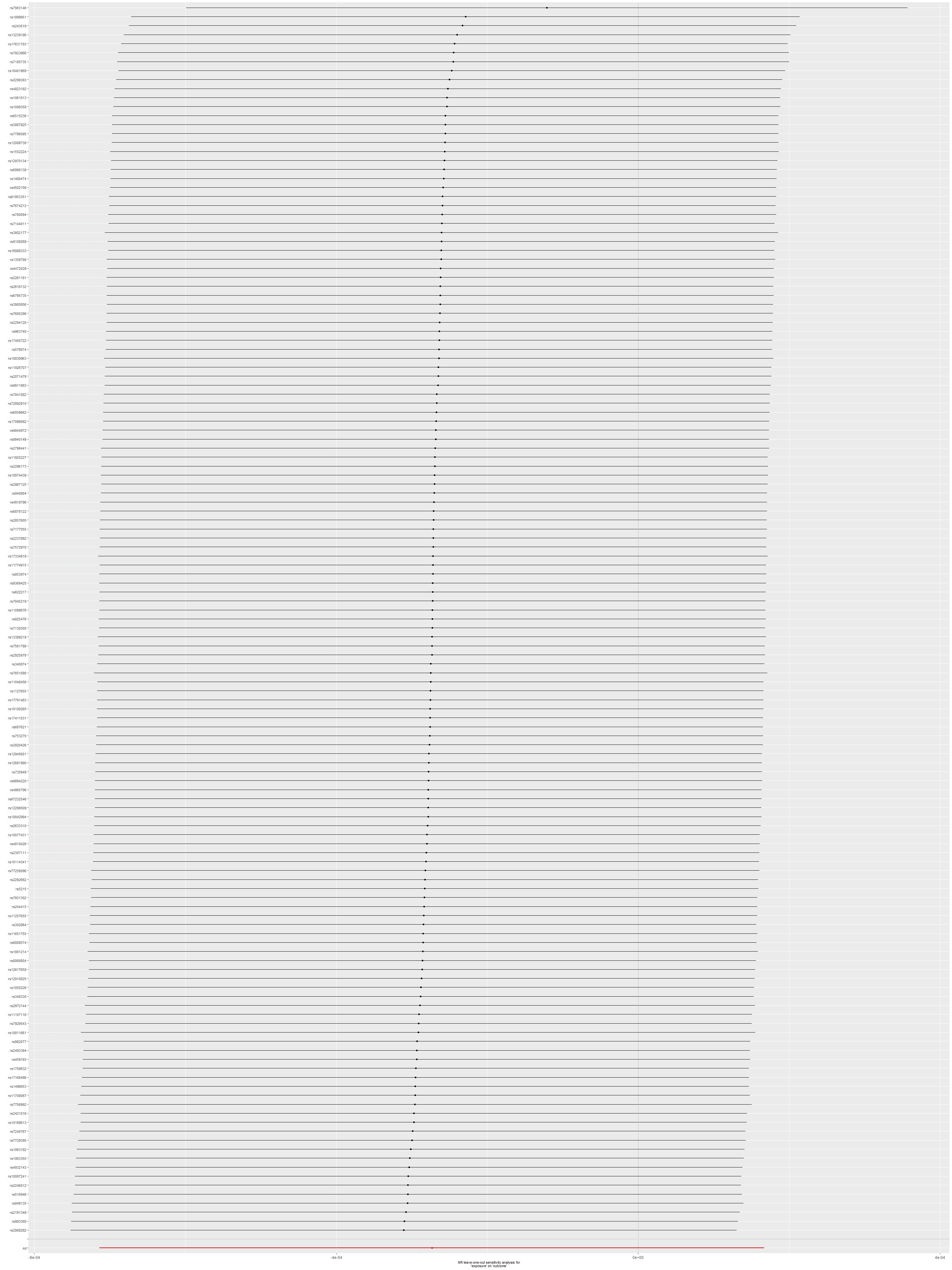

Supplement: Supplementary Figure 3 — MR leave-one-out sensitivity analysis for “T2DM” on “UTI”. MR, Mendelian randomization; T2DM, type 2 diabetes mellitus; UTI, urinary tract infections. [file Image_3.JPEG]

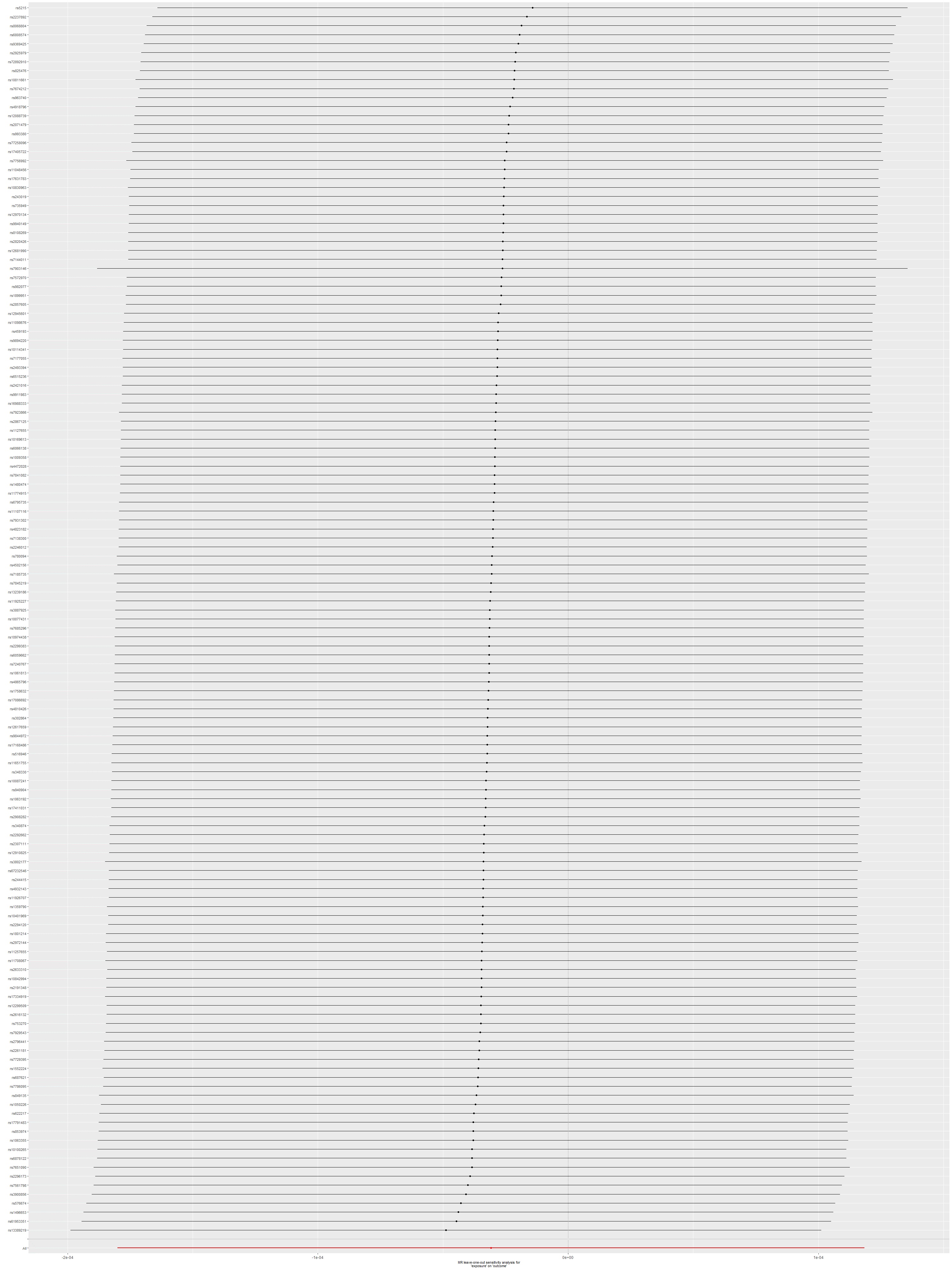

Supplement: Supplementary Figure 4 — MR leave-one-out sensitivity analysis for “T2DM” on “pneumonia”. MR, Mendelian randomization; T2DM, type 2 diabetes mellitus. [file Image_4.JPEG]

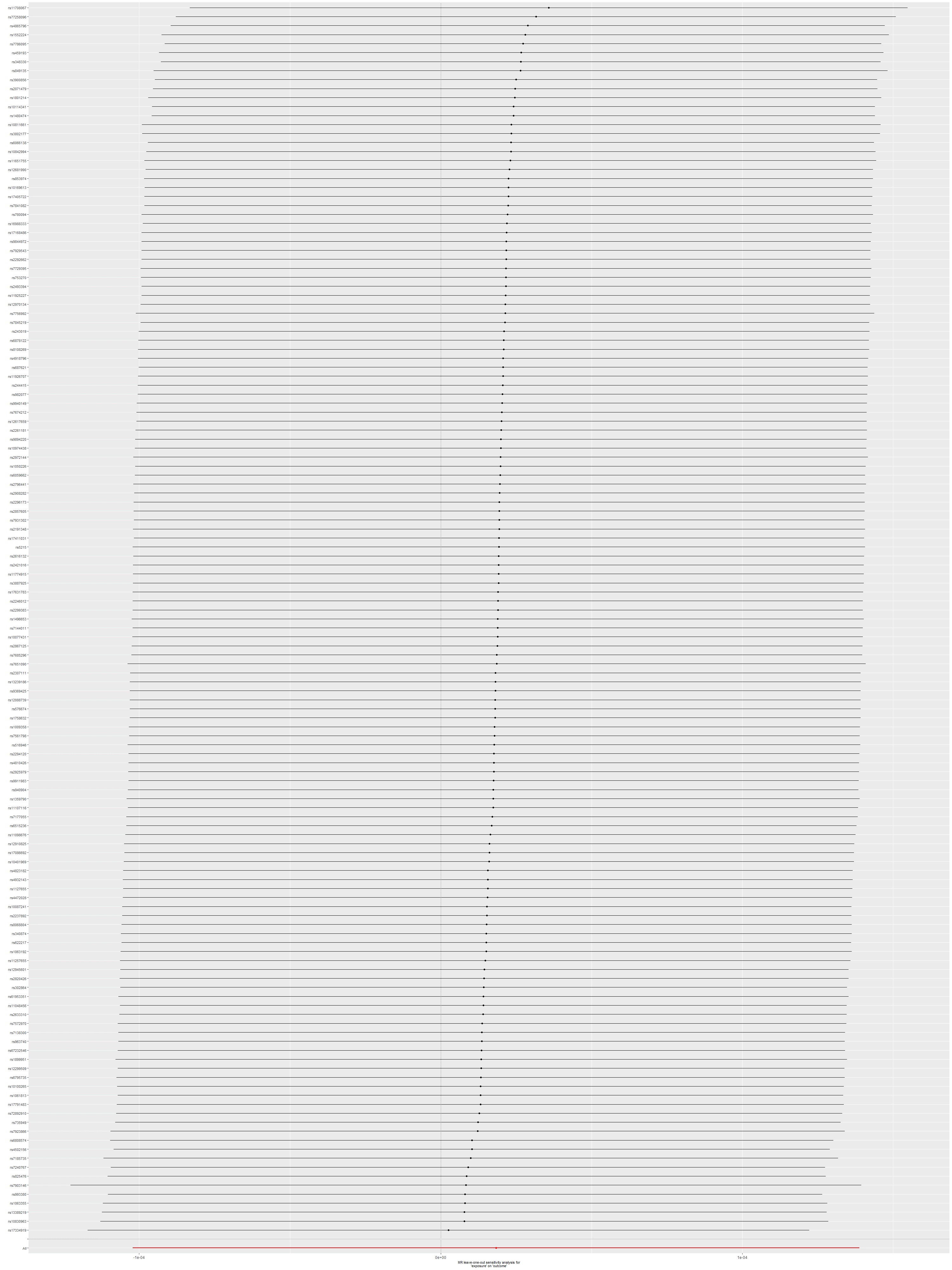

Supplement: Supplementary Figure 5 — MR leave-one-out sensitivity analysis for “T2DM” on “GUI in pregnancy”. MR, Mendelian randomization; T2DM, type 2 diabetes mellitus; GUI, genito-urinary infection. [file Image_5.JPEG]
