# Supplementary material for: No Casual Relationship Between T2DM and the Risk of Infectious Diseases: A Two-Sample Mendelian Randomization Study
Source: Front Genet. 2021 Aug 30;12:720874. doi: 10.3389/fgene.2021.720874 (PMC8435717; doi:10.3389/fgene.2021.720874)
Supplement: Supplementary Table 1 — Characteristics of 137 genetic variants in T2DM and infections datasets. T2DM, type 2 diabetes mellitus; SNP, single-nucleotide polymorphism; Chr, chromosome; EA, effect allele; NEA, non-effect allele; SE, standard error; SSTI, skin and soft tissue infection; UTI, urinary tract infection; GUI, genito-urinary infection. [file Table_1.docx]

**Supplementary Table 1.**  Characteristics of 137 genetic variants in T2DM and infections datasets.

|  |  |  |  | T2DM | | |  | SEPSIS | | |  | SSTI | | |  | UTI | | |  | pneumonia | | |  | GUI | | |
| --- | --- | --- | --- | --- | --- | --- | --- | --- | --- | --- | --- | --- | --- | --- | --- | --- | --- | --- | --- | --- | --- | --- | --- | --- | --- | --- |
| SNP | Chr | EA | NEA | Beta | SE | P-value |  | Beta | SE | P-value |  | Beta | SE | P-value |  | Beta | SE | P-value |  | Beta | SE | P-value |  | Beta | SE | P-value |
| rs2296173 | 1 | G | A | 0.065 | 0.009 | 7.66×10^-14^ |  | 4.66×10^-4^ | 1.14×10^-3^ | 6.83×10^-1^ |  | -5.67×10^-5^ | 1.06×10^-4^ | 5.94×10^-1^ |  | -6.27×10^-5^ | 1.98×10^-4^ | 7.51×10^-1^ |  | 9.72×10^-5^ | 6.69×10^-5^ | 1.46×10^-1^ |  | -1.16×10^-5^ | 5.05×10^-5^ | 8.19×10^-1^ |
| rs12088739 | 1 | G | A | -0.09 | 0.013 | 9.79×10^-12^ |  | 7.85×10^-4^ | 1.19×10^-3^ | 5.09×10^-1^ |  | -5.32×10^-5^ | 1.51×10^-4^ | 7.25×10^-1^ |  | 3.29×10^-4^ | 2.80×10^-4^ | 2.40×10^-1^ |  | 1.30×10^-4^ | 9.50×10^-5^ | 1.70×10^-1^ |  | -6.06×10^-6^ | 7.17×10^-5^ | 9.33×10^-1^ |
| rs1127655 | 1 | C | T | 0.044 | 0.008 | 2.47×10^-08^ |  | -1.74×10^-3^ | 1.38×10^-3^ | 2.08×10^-1^ |  | -7.77×10^-5^ | 8.71×10^-5^ | 3.72×10^-1^ |  | 1.09×10^-5^ | 1.61×10^-4^ | 9.46×10^-1^ |  | -2.09×10^-5^ | 5.47×10^-5^ | 7.03×10^-1^ |  | 3.31×10^-5^ | 4.13×10^-5^ | 4.24×10^-1^ |
| rs2493394 | 1 | G | A | 0.073 | 0.011 | 1.15×10^-10^ |  | -3.12×10^-4^ | 1.33×10^-3^ | 8.14×10^-1^ |  | 5.69×10^-5^ | 1.39×10^-4^ | 6.83×10^-1^ |  | 3.44×10^-4^ | 2.59×10^-4^ | 1.83×10^-1^ |  | -4.71×10^-5^ | 8.76×10^-5^ | 5.91×10^-1^ |  | -5.77×10^-5^ | 6.62×10^-5^ | 3.83×10^-1^ |
| rs340874 | 1 | T | C | -0.06 | 0.007 | 8.41×10^-18^ |  | 5.85×10^-4^ | 9.74×10^-4^ | 5.49×10^-1^ |  | 5.99×10^-5^ | 8.76×10^-5^ | 4.95×10^-1^ |  | 6.05×10^-6^ | 1.63×10^-4^ | 9.70×10^-1^ |  | -2.00×10^-5^ | 5.51×10^-5^ | 7.16×10^-1^ |  | -2.77×10^-5^ | 4.16×10^-5^ | 5.05×10^-1^ |
| rs2820426 | 1 | A | G | -0.05 | 0.007 | 1.30×10^-12^ |  | -1.94×10^-3^ | 1.19×10^-3^ | 1.03×10^-1^ |  | 1.97×10^-4^ | 8.91×10^-5^ | 2.69×10^-2^ |  | -1.77×10^-5^ | 1.65×10^-4^ | 9.15×10^-1^ |  | 5.02×10^-5^ | 5.60×10^-5^ | 3.69×10^-1^ |  | -4.19×10^-5^ | 4.23×10^-5^ | 3.21×10^-1^ |
| rs348330 | 1 | G | A | 0.049 | 0.008 | 1.86×10^-09^ |  | 4.46×10^-6^ | 1.29×10^-3^ | 9.97×10^-1^ |  | -5.08×10^-5^ | 9.05×10^-5^ | 5.75×10^-1^ |  | 1.56×10^-4^ | 1.68×10^-4^ | 3.52×10^-1^ |  | 1.88×10^-5^ | 5.69×10^-5^ | 7.40×10^-1^ |  | -9.25×10^-5^ | 4.29×10^-5^ | 3.12×10^-2^ |
| rs2867125 | 2 | T | C | -0.06 | 0.01 | 4.32×10^-10^ |  | -4.63×10^-4^ | 1.33×10^-3^ | 7.28×10^-1^ |  | -6.26×10^-5^ | 1.15×10^-4^ | 5.86×10^-1^ |  | 6.87×10^-5^ | 2.13×10^-4^ | 7.47×10^-1^ |  | 2.79×10^-5^ | 7.22×10^-5^ | 6.99×10^-1^ |  | 4.78×10^-6^ | 5.45×10^-5^ | 9.30×10^-1^ |
| rs780094 | 2 | T | C | -0.07 | 0.007 | 5.16×10^-21^ |  | 6.88×10^-4^ | 8.99×10^-4^ | 4.44×10^-1^ |  | 2.06×10^-4^ | 8.93×10^-5^ | 2.11×10^-2^ |  | 1.27×10^-4^ | 1.66×10^-4^ | 4.42×10^-1^ |  | 5.26×10^-6^ | 5.61×10^-5^ | 9.25×10^-1^ |  | 2.81×10^-5^ | 4.24×10^-5^ | 5.08×10^-1^ |
| rs17334919 | 2 | T | C | -0.14 | 0.013 | 6.69×10^-28^ |  | -5.34×10^-4^ | 7.15×10^-4^ | 4.55×10^-1^ |  | -8.41×10^-5^ | 1.44×10^-4^ | 5.60×10^-1^ |  | 5.41×10^-5^ | 2.68×10^-4^ | 8.40×10^-1^ |  | -3.52×10^-5^ | 9.07×10^-5^ | 6.98×10^-1^ |  | -1.60×10^-4^ | 6.85×10^-5^ | 1.92×10^-2^ |
| rs243019 | 2 | C | T | 0.057 | 0.007 | 2.29×10^-15^ |  | -6.54×10^-4^ | 1.08×10^-3^ | 5.43×10^-1^ |  | -1.31×10^-4^ | 8.75×10^-5^ | 1.33×10^-1^ |  | -3.87×10^-4^ | 1.62×10^-4^ | 1.71×10^-2^ |  | -4.77×10^-5^ | 5.50×10^-5^ | 3.85×10^-1^ |  | -2.35×10^-5^ | 4.15×10^-5^ | 5.71×10^-1^ |
| rs1009358 | 2 | C | T | -0.05 | 0.008 | 9.81×10^-12^ |  | 1.42×10^-3^ | 1.14×10^-3^ | 2.14×10^-1^ |  | 5.21×10^-5^ | 8.96×10^-5^ | 5.61×10^-1^ |  | 2.13×10^-4^ | 1.66×10^-4^ | 1.99×10^-1^ |  | 1.73×10^-5^ | 5.63×10^-5^ | 7.59×10^-1^ |  | -5.83×10^-6^ | 4.25×10^-5^ | 8.91×10^-1^ |
| rs10169613 | 2 | T | C | -0.04 | 0.008 | 3.57×10^-08^ |  | -2.13×10^-3^ | 1.41×10^-3^ | 1.32×10^-1^ |  | 6.65×10^-5^ | 8.70×10^-5^ | 4.45×10^-1^ |  | -2.74×10^-4^ | 1.61×10^-4^ | 8.98×10^-2^ |  | 2.12×10^-5^ | 5.47×10^-5^ | 6.99×10^-1^ |  | 4.86×10^-5^ | 4.13×10^-5^ | 2.39×10^-1^ |
| rs12617659 | 2 | T | C | -0.07 | 0.01 | 2.83×10^-11^ |  | 1.04×10^-3^ | 1.24×10^-3^ | 4.01×10^-1^ |  | -7.10×10^-5^ | 1.22×10^-4^ | 5.62×10^-1^ |  | -1.73×10^-4^ | 2.27×10^-4^ | 4.45×10^-1^ |  | -1.88×10^-5^ | 7.68×10^-5^ | 8.07×10^-1^ |  | 2.60×10^-5^ | 5.80×10^-5^ | 6.54×10^-1^ |
| rs7572970 | 2 | A | G | -0.06 | 0.009 | 1.39×10^-11^ |  | -2.00×10^-4^ | 1.14×10^-3^ | 8.61×10^-1^ |  | 3.90×10^-5^ | 9.68×10^-5^ | 6.87×10^-1^ |  | 3.43×10^-5^ | 1.80×10^-4^ | 8.49×10^-1^ |  | 4.66×10^-5^ | 6.08×10^-5^ | 4.44×10^-1^ |  | -5.09×10^-5^ | 4.60×10^-5^ | 2.68×10^-1^ |
| rs13389219 | 2 | T | C | -0.07 | 0.007 | 2.11×10^-22^ |  | 9.85×10^-4^ | 8.57×10^-4^ | 2.51×10^-1^ |  | -5.13×10^-5^ | 8.89×10^-5^ | 5.64×10^-1^ |  | 2.24×10^-5^ | 1.65×10^-4^ | 8.92×10^-1^ |  | -1.31×10^-4^ | 5.59×10^-5^ | 1.91×10^-2^ |  | -7.89×10^-5^ | 4.22×10^-5^ | 6.16×10^-2^ |
| rs2972144 | 2 | A | G | -0.09 | 0.008 | 2.55×10^-34^ |  | 2.52×10^-4^ | 6.92×10^-4^ | 7.16×10^-1^ |  | 4.22×10^-5^ | 9.08×10^-5^ | 6.42×10^-1^ |  | -7.08×10^-5^ | 1.68×10^-4^ | 6.74×10^-1^ |  | -1.90×10^-5^ | 5.71×10^-5^ | 7.39×10^-1^ |  | 7.57×10^-6^ | 4.31×10^-5^ | 8.61×10^-1^ |
| rs7561798 | 2 | G | A | 0.04 | 0.007 | 2.79×10^-08^ |  | -3.85×10^-4^ | 1.51×10^-3^ | 7.99×10^-1^ |  | -3.86×10^-5^ | 8.69×10^-5^ | 6.57×10^-1^ |  | -1.28×10^-5^ | 1.61×10^-4^ | 9.37×10^-1^ |  | 1.17×10^-4^ | 5.46×10^-5^ | 3.20×10^-2^ |  | 7.07×10^-6^ | 4.12×10^-5^ | 8.64×10^-1^ |
| rs1899951 | 3 | T | C | -0.11 | 0.011 | 1.64×10^-24^ |  | 4.39×10^-4^ | 8.25×10^-4^ | 5.94×10^-1^ |  | 2.16×10^-6^ | 1.32×10^-4^ | 9.87×10^-1^ |  | 5.03×10^-4^ | 2.46×10^-4^ | 4.06×10^-2^ |  | 4.66×10^-5^ | 8.32×10^-5^ | 5.75×10^-1^ |  | -5.38×10^-5^ | 6.29×10^-5^ | 3.92×10^-1^ |
| rs1496653 | 3 | G | A | -0.08 | 0.009 | 2.57×10^-18^ |  | -4.23×10^-4^ | 9.74×10^-4^ | 6.64×10^-1^ |  | 9.89×10^-5^ | 1.08×10^-4^ | 3.58×10^-1^ |  | -2.06×10^-4^ | 2.00×10^-4^ | 3.02×10^-1^ |  | -1.30×10^-4^ | 6.76×10^-5^ | 5.52×10^-2^ |  | 4.36×10^-6^ | 5.11×10^-5^ | 9.32×10^-1^ |
| rs11926707 | 3 | T | C | -0.05 | 0.008 | 1.69×10^-08^ |  | 3.09×10^-4^ | 1.35×10^-3^ | 8.20×10^-1^ |  | 1.36×10^-4^ | 9.01×10^-5^ | 1.32×10^-1^ |  | 1.17×10^-4^ | 1.67×10^-4^ | 4.83×10^-1^ |  | -3.60×10^-5^ | 5.66×10^-5^ | 5.24×10^-1^ |  | 2.66×10^-5^ | 4.28×10^-5^ | 5.34×10^-1^ |
| rs2292662 | 3 | T | C | -0.06 | 0.011 | 1.24×10^-08^ |  | 1.33×10^-3^ | 1.35×10^-3^ | 3.25×10^-1^ |  | -3.45×10^-6^ | 1.22×10^-4^ | 9.77×10^-1^ |  | -1.30×10^-4^ | 2.26×10^-4^ | 5.66×10^-1^ |  | -4.21×10^-5^ | 7.67×10^-5^ | 5.83×10^-1^ |  | 5.22×10^-5^ | 5.79×10^-5^ | 3.67×10^-1^ |
| rs6795735 | 3 | T | C | -0.06 | 0.007 | 1.63×10^-14^ |  | 8.33×10^-5^ | 1.10×10^-3^ | 9.40×10^-1^ |  | 2.25×10^-5^ | 8.85×10^-5^ | 8.00×10^-1^ |  | 1.19×10^-4^ | 1.64×10^-4^ | 4.68×10^-1^ |  | 1.15×10^-5^ | 5.56×10^-5^ | 8.36×10^-1^ |  | -4.92×10^-5^ | 4.20×10^-5^ | 2.42×10^-1^ |
| rs11708067 | 3 | G | A | -0.1 | 0.009 | 5.93×10^-29^ |  | 1.69×10^-4^ | 7.27×10^-4^ | 8.16×10^-1^ |  | -1.92×10^-4^ | 1.01×10^-4^ | 5.71×10^-2^ |  | -1.31×10^-4^ | 1.87×10^-4^ | 4.83×10^-1^ |  | -2.37×10^-5^ | 6.34×10^-5^ | 7.09×10^-1^ |  | 1.22×10^-4^ | 4.79×10^-5^ | 1.11×10^-2^ |
| rs9844972 | 3 | C | G | 0.096 | 0.015 | 1.03×10^-10^ |  | 1.45×10^-4^ | 1.24×10^-3^ | 9.07×10^-1^ |  | -3.26×10^-5^ | 1.72×10^-4^ | 8.49×10^-1^ |  | -1.35×10^-4^ | 3.18×10^-4^ | 6.71×10^-1^ |  | 2.93×10^-5^ | 1.08×10^-4^ | 7.86×10^-1^ |  | -6.90×10^-5^ | 8.15×10^-5^ | 3.97×10^-1^ |
| rs4472028 | 3 | T | C | 0.045 | 0.007 | 2.08×10^-10^ |  | -3.71×10^-4^ | 1.34×10^-3^ | 7.82×10^-1^ |  | -1.59×10^-4^ | 8.72×10^-5^ | 6.75×10^-2^ |  | -1.43×10^-4^ | 1.62×10^-4^ | 3.78×10^-1^ |  | -1.82×10^-5^ | 5.48×10^-5^ | 7.40×10^-1^ |  | 3.34×10^-5^ | 4.14×10^-5^ | 4.20×10^-1^ |
| rs11925227 | 3 | A | G | -0.05 | 0.01 | 2.25×10^-08^ |  | -1.67×10^-4^ | 1.46×10^-3^ | 9.09×10^-1^ |  | -6.96×10^-5^ | 1.12×10^-4^ | 5.35×10^-1^ |  | 7.71×10^-5^ | 2.08×10^-4^ | 7.11×10^-1^ |  | -4.69×10^-6^ | 7.04×10^-5^ | 9.47×10^-1^ |  | 5.01×10^-5^ | 5.32×10^-5^ | 3.46×10^-1^ |
| rs7651090 | 3 | G | A | 0.12 | 0.008 | 3.85×10^-57^ |  | 5.81×10^-4^ | 5.41×10^-4^ | 2.83×10^-1^ |  | -2.02×10^-4^ | 9.36×10^-5^ | 3.09×10^-2^ |  | -2.57×10^-5^ | 1.74×10^-4^ | 8.82×10^-1^ |  | 3.33×10^-5^ | 5.88×10^-5^ | 5.72×10^-1^ |  | 1.18×10^-6^ | 4.44×10^-5^ | 9.79×10^-1^ |
| rs3887925 | 3 | C | T | -0.05 | 0.008 | 2.47×10^-09^ |  | 9.05×10^-4^ | 1.28×10^-3^ | 4.80×10^-1^ |  | 9.87×10^-5^ | 8.74×10^-5^ | 2.59×10^-1^ |  | 2.08×10^-4^ | 1.62×10^-4^ | 1.99×10^-1^ |  | -4.25×10^-6^ | 5.49×10^-5^ | 9.38×10^-1^ |  | 7.96×10^-6^ | 4.15×10^-5^ | 8.48×10^-1^ |
| rs6808574 | 3 | T | C | -0.06 | 0.008 | 4.38×10^-13^ |  | 3.77×10^-4^ | 1.13×10^-3^ | 7.38×10^-1^ |  | 9.63×10^-6^ | 8.94×10^-5^ | 9.14×10^-1^ |  | -9.93×10^-5^ | 1.66×10^-4^ | 5.49×10^-1^ |  | 1.14×10^-4^ | 5.62×10^-5^ | 4.19×10^-2^ |  | -7.93×10^-5^ | 4.24×10^-5^ | 6.14×10^-2^ |
| rs1801214 | 4 | C | T | -0.09 | 0.007 | 5.52×10^-34^ |  | 9.93×10^-4^ | 6.83×10^-4^ | 1.46×10^-1^ |  | 1.61×10^-5^ | 8.87×10^-5^ | 8.56×10^-1^ |  | -4.52×10^-5^ | 1.64×10^-4^ | 7.84×10^-1^ |  | -1.73×10^-5^ | 5.57×10^-5^ | 7.57×10^-1^ |  | 3.47×10^-5^ | 4.21×10^-5^ | 4.10×10^-1^ |
| rs17086692 | 4 | T | G | -0.05 | 0.008 | 2.48×10^-08^ |  | 7.15×10^-4^ | 1.40×10^-3^ | 6.10×10^-1^ |  | 1.06×10^-4^ | 9.40×10^-5^ | 2.61×10^-1^ |  | 8.69×10^-5^ | 1.74×10^-4^ | 6.18×10^-1^ |  | -1.39×10^-5^ | 5.90×10^-5^ | 8.13×10^-1^ |  | -2.93×10^-5^ | 4.46×10^-5^ | 5.12×10^-1^ |
| rs993380 | 4 | A | G | 0.051 | 0.008 | 4.59×10^-10^ |  | -8.21×10^-4^ | 1.26×10^-3^ | 5.16×10^-1^ |  | -4.04×10^-5^ | 9.21×10^-5^ | 6.61×10^-1^ |  | 3.98×10^-4^ | 1.71×10^-4^ | 1.98×10^-2^ |  | -8.02×10^-5^ | 5.79×10^-5^ | 1.66×10^-1^ |  | 1.17×10^-4^ | 4.37×10^-5^ | 7.59×10^-3^ |
| rs7674212 | 4 | T | G | -0.05 | 0.008 | 6.18×10^-10^ |  | -1.28×10^-3^ | 1.33×10^-3^ | 3.35×10^-1^ |  | -6.80×10^-5^ | 8.88×10^-5^ | 4.44×10^-1^ |  | 1.75×10^-4^ | 1.65×10^-4^ | 2.88×10^-1^ |  | 1.07×10^-4^ | 5.58×10^-5^ | 5.57×10^-2^ |  | 2.05×10^-5^ | 4.21×10^-5^ | 6.26×10^-1^ |
| rs11098676 | 4 | T | C | -0.05 | 0.01 | 2.03×10^-08^ |  | -3.26×10^-3^ | 1.38×10^-3^ | 1.83×10^-2^ |  | -2.80×10^-5^ | 1.07×10^-4^ | 7.94×10^-1^ |  | 2.61×10^-5^ | 1.99×10^-4^ | 8.96×10^-1^ |  | 4.27×10^-5^ | 6.73×10^-5^ | 5.26×10^-1^ |  | -2.75×10^-5^ | 5.09×10^-5^ | 5.88×10^-1^ |
| rs7685296 | 4 | T | C | -0.05 | 0.008 | 2.32×10^-10^ |  | -9.71×10^-4^ | 1.32×10^-3^ | 4.63×10^-1^ |  | -9.70×10^-5^ | 9.72×10^-5^ | 3.18×10^-1^ |  | 1.50×10^-4^ | 1.80×10^-4^ | 4.04×10^-1^ |  | -6.11×10^-6^ | 6.10×10^-5^ | 9.20×10^-1^ |  | 2.08×10^-6^ | 4.61×10^-5^ | 9.64×10^-1^ |
| rs735949 | 4 | C | T | -0.07 | 0.011 | 1.95×10^-11^ |  | 1.30×10^-3^ | 1.23×10^-3^ | 2.88×10^-1^ |  | 9.16×10^-5^ | 1.25×10^-4^ | 4.65×10^-1^ |  | -4.41×10^-5^ | 2.32×10^-4^ | 8.49×10^-1^ |  | 7.70×10^-5^ | 7.87×10^-5^ | 3.27×10^-1^ |  | -9.08×10^-5^ | 5.94×10^-5^ | 1.27×10^-1^ |
| rs1061813 | 5 | G | A | 0.043 | 0.007 | 3.37×10^-09^ |  | 8.97×10^-4^ | 1.41×10^-3^ | 5.26×10^-1^ |  | -3.80×10^-5^ | 8.72×10^-5^ | 6.63×10^-1^ |  | -2.54×10^-4^ | 1.62×10^-4^ | 1.16×10^-1^ |  | 8.37×10^-6^ | 5.48×10^-5^ | 8.79×10^-1^ |  | 6.28×10^-5^ | 4.14×10^-5^ | 1.29×10^-1^ |
| rs4865796 | 5 | G | A | -0.05 | 0.008 | 1.33×10^-11^ |  | -4.92×10^-4^ | 1.23×10^-3^ | 6.90×10^-1^ |  | -5.38×10^-5^ | 9.40×10^-5^ | 5.67×10^-1^ |  | -3.82×10^-5^ | 1.74×10^-4^ | 8.27×10^-1^ |  | -8.94×10^-6^ | 5.90×10^-5^ | 8.80×10^-1^ |  | 1.18×10^-4^ | 4.46×10^-5^ | 8.32×10^-3^ |
| rs459193 | 5 | A | G | -0.07 | 0.008 | 8.81×10^-18^ |  | 4.98×10^-4^ | 1.03×10^-3^ | 6.28×10^-1^ |  | -6.45×10^-5^ | 1.05×10^-4^ | 5.39×10^-1^ |  | -1.92×10^-4^ | 1.95×10^-4^ | 3.23×10^-1^ |  | 3.10×10^-5^ | 6.60×10^-5^ | 6.39×10^-1^ |  | 8.61×10^-5^ | 4.98×10^-5^ | 8.39×10^-2^ |
| rs3900856 | 5 | A | G | 0.114 | 0.019 | 7.35×10^-10^ |  | 1.45×10^-3^ | 1.39×10^-3^ | 2.96×10^-1^ |  | 9.80×10^-6^ | 2.27×10^-4^ | 9.65×10^-1^ |  | -3.65×10^-4^ | 4.20×10^-4^ | 3.86×10^-1^ |  | 3.01×10^-4^ | 1.42×10^-4^ | 3.46×10^-2^ |  | -2.03×10^-4^ | 1.08×10^-4^ | 5.94×10^-2^ |
| rs2307111 | 5 | C | T | -0.04 | 0.007 | 3.03×10^-08^ |  | 1.22×10^-3^ | 1.52×10^-3^ | 4.23×10^-1^ |  | 3.13×10^-5^ | 8.90×10^-5^ | 7.25×10^-1^ |  | -8.55×10^-5^ | 1.65×10^-4^ | 6.04×10^-1^ |  | -3.66×10^-5^ | 5.59×10^-5^ | 5.13×10^-1^ |  | -2.99×10^-6^ | 4.22×10^-5^ | 9.44×10^-1^ |
| rs6878122 | 5 | G | A | 0.056 | 0.008 | 1.19×10^-12^ |  | -1.59×10^-4^ | 1.15×10^-3^ | 8.90×10^-1^ |  | -5.44×10^-5^ | 9.32×10^-5^ | 5.60×10^-1^ |  | -4.03×10^-5^ | 1.73×10^-4^ | 8.16×10^-1^ |  | 7.82×10^-5^ | 5.86×10^-5^ | 1.82×10^-1^ |  | -2.56×10^-5^ | 4.42×10^-5^ | 5.62×10^-1^ |
| rs7729395 | 5 | T | C | 0.137 | 0.016 | 1.10×10^-17^ |  | 7.18×10^-4^ | 9.76×10^-4^ | 4.62×10^-1^ |  | 1.82×10^-4^ | 1.93×10^-4^ | 3.45×10^-1^ |  | 4.44×10^-4^ | 3.58×10^-4^ | 2.15×10^-1^ |  | 8.64×10^-5^ | 1.21×10^-4^ | 4.76×10^-1^ |  | -5.83×10^-5^ | 9.16×10^-5^ | 5.25×10^-1^ |
| rs10077431 | 5 | A | C | -0.05 | 0.009 | 4.76×10^-08^ |  | -2.11×10^-3^ | 1.51×10^-3^ | 1.61×10^-1^ |  | -7.91×10^-5^ | 1.05×10^-4^ | 4.53×10^-1^ |  | -9.07×10^-5^ | 1.96×10^-4^ | 6.43×10^-1^ |  | -6.87×10^-6^ | 6.63×10^-5^ | 9.17×10^-1^ |  | 7.06×10^-6^ | 5.00×10^-5^ | 8.88×10^-1^ |
| rs1050226 | 6 | G | A | -0.05 | 0.007 | 3.34×10^-11^ |  | -4.89×10^-4^ | 1.25×10^-3^ | 6.96×10^-1^ |  | 1.11×10^-4^ | 8.82×10^-5^ | 2.06×10^-1^ |  | -1.44×10^-4^ | 1.64×10^-4^ | 3.79×10^-1^ |  | -6.51×10^-5^ | 5.54×10^-5^ | 2.40×10^-1^ |  | 1.50×10^-5^ | 4.19×10^-5^ | 7.20×10^-1^ |
| rs7756992 | 6 | G | A | 0.13 | 0.008 | 6.00×10^-62^ |  | -1.26×10^-4^ | 5.27×10^-4^ | 8.11×10^-1^ |  | 2.90×10^-5^ | 9.84×10^-5^ | 7.68×10^-1^ |  | 7.51×10^-5^ | 1.82×10^-4^ | 6.81×10^-1^ |  | -3.12×10^-5^ | 6.18×10^-5^ | 6.14×10^-1^ |  | -1.26×10^-5^ | 4.67×10^-5^ | 7.87×10^-1^ |
| rs2857605 | 6 | C | T | -0.07 | 0.009 | 5.90×10^-14^ |  | -1.55×10^-3^ | 1.10×10^-3^ | 1.60×10^-1^ |  | -1.98×10^-5^ | 1.06×10^-4^ | 8.52×10^-1^ |  | 4.19×10^-5^ | 1.97×10^-4^ | 8.32×10^-1^ |  | 4.61×10^-5^ | 6.69×10^-5^ | 4.90×10^-1^ |  | 1.07×10^-5^ | 5.05×10^-5^ | 8.32×10^-1^ |
| rs1063355 | 6 | T | G | -0.07 | 0.008 | 3.72×10^-19^ |  | -1.19×10^-5^ | 8.74×10^-4^ | 9.89×10^-1^ |  | 2.18×10^-4^ | 8.91×10^-5^ | 1.42×10^-2^ |  | -2.00×10^-4^ | 1.65×10^-4^ | 2.26×10^-1^ |  | -5.18×10^-5^ | 5.60×10^-5^ | 3.55×10^-1^ |  | -7.87×10^-5^ | 4.23×10^-5^ | 6.27×10^-2^ |
| rs2071479 | 6 | T | C | 0.147 | 0.023 | 6.62×10^-11^ |  | -5.02×10^-5^ | 1.26×10^-3^ | 9.68×10^-1^ |  | 3.27×10^-4^ | 2.67×10^-4^ | 2.20×10^-1^ |  | -3.17×10^-4^ | 4.95×10^-4^ | 5.22×10^-1^ |  | -2.33×10^-4^ | 1.68×10^-4^ | 1.65×10^-1^ |  | -2.07×10^-4^ | 1.27×10^-4^ | 1.01×10^-1^ |
| rs9369425 | 6 | G | A | 0.055 | 0.009 | 1.13×10^-10^ |  | -1.29×10^-3^ | 1.22×10^-3^ | 2.88×10^-1^ |  | -7.14×10^-5^ | 9.54×10^-5^ | 4.54×10^-1^ |  | -2.75×10^-5^ | 1.77×10^-4^ | 8.76×10^-1^ |  | -1.25×10^-4^ | 5.99×10^-5^ | 3.65×10^-2^ |  | 3.66×10^-6^ | 4.53×10^-5^ | 9.36×10^-1^ |
| rs72892910 | 6 | T | G | 0.065 | 0.01 | 6.43×10^-11^ |  | -1.24×10^-4^ | 1.24×10^-3^ | 9.21×10^-1^ |  | -3.11×10^-5^ | 1.16×10^-4^ | 7.88×10^-1^ |  | -1.07×10^-4^ | 2.14×10^-4^ | 6.18×10^-1^ |  | -1.36×10^-4^ | 7.26×10^-5^ | 6.10×10^-2^ |  | 7.86×10^-5^ | 5.48×10^-5^ | 1.52×10^-1^ |
| rs853974 | 6 | T | C | 0.06 | 0.009 | 7.86×10^-12^ |  | -1.48×10^-3^ | 1.15×10^-3^ | 1.98×10^-1^ |  | 6.58×10^-5^ | 9.91×10^-5^ | 5.07×10^-1^ |  | -3.16×10^-5^ | 1.84×10^-4^ | 8.64×10^-1^ |  | 7.68×10^-5^ | 6.23×10^-5^ | 2.17×10^-1^ |  | -4.49×10^-5^ | 4.70×10^-5^ | 3.40×10^-1^ |
| rs2246012 | 6 | C | T | 0.053 | 0.009 | 2.43×10^-08^ |  | 2.83×10^-3^ | 1.53×10^-3^ | 6.52×10^-2^ |  | -1.02×10^-4^ | 1.16×10^-4^ | 3.80×10^-1^ |  | 5.33×10^-4^ | 2.15×10^-4^ | 1.33×10^-2^ |  | -1.37×10^-5^ | 7.30×10^-5^ | 8.51×10^-1^ |  | -9.84×10^-6^ | 5.51×10^-5^ | 8.58×10^-1^ |
| rs622217 | 6 | C | T | -0.05 | 0.008 | 3.13×10^-10^ |  | 5.20×10^-4^ | 1.25×10^-3^ | 6.78×10^-1^ |  | 3.77×10^-5^ | 8.71×10^-5^ | 6.65×10^-1^ |  | 2.37×10^-5^ | 1.61×10^-4^ | 8.83×10^-1^ |  | -7.08×10^-5^ | 5.47×10^-5^ | 1.96×10^-1^ |  | -3.48×10^-5^ | 4.13×10^-5^ | 4.00×10^-1^ |
| rs17168486 | 7 | T | C | 0.074 | 0.009 | 2.18×10^-15^ |  | -1.05×10^-3^ | 1.08×10^-3^ | 3.31×10^-1^ |  | 1.14×10^-5^ | 1.15×10^-4^ | 9.21×10^-1^ |  | 2.42×10^-4^ | 2.14×10^-4^ | 2.58×10^-1^ |  | 1.65×10^-5^ | 7.24×10^-5^ | 8.19×10^-1^ |  | -4.13×10^-5^ | 5.47×10^-5^ | 4.50×10^-1^ |
| rs2191348 | 7 | G | T | -0.07 | 0.007 | 3.44×10^-19^ |  | -9.77×10^-4^ | 9.31×10^-4^ | 2.94×10^-1^ |  | -1.20×10^-4^ | 8.72×10^-5^ | 1.68×10^-1^ |  | -2.53×10^-4^ | 1.62×10^-4^ | 1.18×10^-1^ |  | -2.84×10^-5^ | 5.48×10^-5^ | 6.04×10^-1^ |  | 6.81×10^-6^ | 4.14×10^-5^ | 8.69×10^-1^ |
| rs849135 | 7 | G | A | 0.1 | 0.007 | 1.04×10^-43^ |  | -4.94×10^-4^ | 6.06×10^-4^ | 4.14×10^-1^ |  | 2.25×10^-5^ | 8.69×10^-5^ | 7.96×10^-1^ |  | 1.34×10^-4^ | 1.61×10^-4^ | 4.05×10^-1^ |  | 2.64×10^-5^ | 5.46×10^-5^ | 6.28×10^-1^ |  | -3.92×10^-5^ | 4.12×10^-5^ | 3.42×10^-1^ |
| rs2908282 | 7 | A | G | 0.055 | 0.009 | 4.25×10^-09^ |  | 2.59×10^-3^ | 1.43×10^-3^ | 7.03×10^-2^ |  | -7.15×10^-5^ | 1.14×10^-4^ | 5.29×10^-1^ |  | 5.80×10^-4^ | 2.11×10^-4^ | 5.89×10-3 |  | 3.47×10^-5^ | 7.13×10^-5^ | 6.26×10^-1^ |  | -1.70×10^-5^ | 5.39×10^-5^ | 7.53×10^-1^ |
| rs2299383 | 7 | T | C | 0.041 | 0.007 | 1.49×10^-08^ |  | -1.84×10^-4^ | 1.49×10^-3^ | 9.02×10^-1^ |  | 1.40×10^-4^ | 8.82×10^-5^ | 1.12×10^-1^ |  | -3.09×10^-4^ | 1.64×10^-4^ | 5.89×10^-2^ |  | 7.71×10^-6^ | 5.54×10^-5^ | 8.89×10^-1^ |  | -7.05×10^-6^ | 4.19×10^-5^ | 8.66×10^-1^ |
| rs13239186 | 7 | T | C | 0.054 | 0.009 | 2.70×10^-10^ |  | -8.76×10^-5^ | 1.23×10^-3^ | 9.43×10^-1^ |  | 5.35×10^-5^ | 9.50×10^-5^ | 5.73×10^-1^ |  | -3.95×10^-4^ | 1.76×10^-4^ | 2.51×10^-2^ |  | 1.96×10-07 | 5.97×10^-5^ | 9.97×10^-1^ |  | 3.18×10^-6^ | 4.51×10^-5^ | 9.44×10^-1^ |
| rs7786095 | 7 | G | A | -0.07 | 0.013 | 9.64×10^-09^ |  | 7.24×10^-4^ | 1.33×10^-3^ | 5.85×10^-1^ |  | 3.15×10^-6^ | 1.42×10^-4^ | 9.82×10^-1^ |  | 3.45×10^-4^ | 2.63×10^-4^ | 1.89×10^-1^ |  | -9.58×10^-5^ | 8.90×10^-5^ | 2.82×10^-1^ |  | 1.63×10^-4^ | 6.72×10^-5^ | 1.51×10^-2^ |
| rs7841082 | 8 | T | C | -0.04 | 0.008 | 4.94×10^-08^ |  | 1.08×10^-3^ | 1.46×10^-3^ | 4.57×10^-1^ |  | -8.39×10^-5^ | 8.80×10^-5^ | 3.40×10^-1^ |  | 9.40×10^-5^ | 1.63×10^-4^ | 5.64×10^-1^ |  | 1.88×10^-5^ | 5.53×10^-5^ | 7.34×10^-1^ |  | 4.90×10^-5^ | 4.17×10^-5^ | 2.40×10^-1^ |
| rs11774915 | 8 | T | C | 0.05 | 0.009 | 8.73×10^-09^ |  | -1.09×10^-3^ | 1.29×10^-3^ | 3.96×10^-1^ |  | 1.40×10^-4^ | 9.19×10^-5^ | 1.28×10^-1^ |  | -3.14×10^-5^ | 1.70×10^-4^ | 8.54×10^-1^ |  | -1.72×10^-5^ | 5.77×10^-5^ | 7.65×10^-1^ |  | -8.46×10^-6^ | 4.36×10^-5^ | 8.46×10^-1^ |
| rs10100265 | 8 | A | C | 0.049 | 0.008 | 6.29×10^-10^ |  | -2.69×10^-3^ | 1.26×10^-3^ | 3.35×10^-2^ |  | -1.74×10^-4^ | 8.92×10^-5^ | 5.14×10^-2^ |  | 1.04×10^-5^ | 1.65×10^-4^ | 9.50×10^-1^ |  | 8.24×10^-5^ | 5.61×10^-5^ | 1.42×10^-1^ |  | 5.70×10^-5^ | 4.23×10^-5^ | 1.79×10^-1^ |
| rs17411031 | 8 | G | C | -0.05 | 0.008 | 3.04×10^-08^ |  | -1.08×10^-3^ | 1.53×10^-3^ | 4.80×10^-1^ |  | 8.42×10^-5^ | 9.88×10^-5^ | 3.94×10^-1^ |  | -2.19×10^-5^ | 1.83×10^-4^ | 9.05×10^-1^ |  | -3.09×10^-5^ | 6.21×10^-5^ | 6.18×10^-1^ |  | 1.39×10^-5^ | 4.69×10^-5^ | 7.68×10^-1^ |
| rs10087241 | 8 | G | A | 0.048 | 0.008 | 2.80×10^-09^ |  | -1.38×10^-4^ | 1.30×10^-3^ | 9.15×10^-1^ |  | 6.72×10^-5^ | 8.86×10^-5^ | 4.48×10^-1^ |  | 3.36×10^-4^ | 1.64×10^-4^ | 4.09×10^-2^ |  | 2.15×10^-5^ | 5.57×10^-5^ | 7.00×10^-1^ |  | 3.50×10^-5^ | 4.21×10^-5^ | 4.05×10^-1^ |
| rs12681990 | 8 | C | T | 0.063 | 0.01 | 3.62×10^-11^ |  | -2.52×10^-5^ | 1.31×10^-3^ | 9.85×10^-1^ |  | -2.05×10^-5^ | 1.19×10^-4^ | 8.63×10^-1^ |  | 4.70×10^-5^ | 2.21×10^-4^ | 8.31×10^-1^ |  | -7.30×10^-5^ | 7.48×10^-5^ | 3.29×10^-1^ |  | -6.61×10^-5^ | 5.65×10^-5^ | 2.42×10^-1^ |
| rs516946 | 8 | T | C | -0.08 | 0.009 | 3.16×10^-22^ |  | -4.98×10^-4^ | 8.62×10^-4^ | 5.64×10^-1^ |  | 6.86×10^-5^ | 1.02×10^-4^ | 5.01×10^-1^ |  | -2.48×10^-4^ | 1.89×10^-4^ | 1.90×10^-1^ |  | -1.09×10^-5^ | 6.41×10^-5^ | 8.65×10^-1^ |  | -6.99×10^-6^ | 4.84×10^-5^ | 8.85×10^-1^ |
| rs7845219 | 8 | C | T | -0.04 | 0.007 | 4.54×10^-09^ |  | -2.39×10^-3^ | 1.43×10^-3^ | 9.50×10^-2^ |  | -3.91×10^-5^ | 8.69×10^-5^ | 6.53×10^-1^ |  | 2.23×10^-5^ | 1.61×10^-4^ | 8.90×10^-1^ |  | 1.38×10^-6^ | 5.46×10^-5^ | 9.80×10^-1^ |  | 3.53×10^-5^ | 4.13×10^-5^ | 3.92×10^-1^ |
| rs3802177 | 8 | A | G | -0.12 | 0.008 | 2.32×10^-52^ |  | -9.61×10^-4^ | 5.37×10^-4^ | 7.32×10^-2^ |  | -1.17×10^-4^ | 9.39×10^-5^ | 2.13×10^-1^ |  | 9.55×10^-5^ | 1.74×10^-4^ | 5.83×10^-1^ |  | -1.10×10^-5^ | 5.90×10^-5^ | 8.53×10^-1^ |  | 2.19×10^-5^ | 4.46×10^-5^ | 6.23×10^-1^ |
| rs2294120 | 8 | G | A | -0.04 | 0.008 | 1.62×10^-08^ |  | -3.07×10^-4^ | 1.37×10^-3^ | 8.23×10^-1^ |  | 8.64×10^-6^ | 8.73×10^-5^ | 9.21×10^-1^ |  | 1.34×10^-4^ | 1.62×10^-4^ | 4.09×10^-1^ |  | -3.81×10^-5^ | 5.48×10^-5^ | 4.88×10^-1^ |  | -8.68×10^-6^ | 4.14×10^-5^ | 8.34×10^-1^ |
| rs10974438 | 9 | C | A | 0.059 | 0.008 | 3.01×10^-15^ |  | 1.74×10^-3^ | 1.07×10^-3^ | 1.04×10^-1^ |  | 7.66×10^-5^ | 9.11×10^-5^ | 4.00×10^-1^ |  | -4.99×10^-5^ | 1.69×10^-4^ | 7.68×10^-1^ |  | 4.78×10^-6^ | 5.72×10^-5^ | 9.33×10^-1^ |  | -1.36×10^-5^ | 4.32×10^-5^ | 7.54×10^-1^ |
| rs1063192 | 9 | G | A | -0.06 | 0.007 | 3.30×10^-18^ |  | -1.83×10^-3^ | 9.59×10^-4^ | 5.64×10^-2^ |  | -7.72×10^-5^ | 8.74×10^-5^ | 3.77×10^-1^ |  | -2.10×10^-4^ | 1.62×10^-4^ | 1.95×10^-1^ |  | -1.51×10^-5^ | 5.49×10^-5^ | 7.83×10^-1^ |  | -2.77×10^-5^ | 4.15×10^-5^ | 5.05×10^-1^ |
| rs10811661 | 9 | C | T | -0.16 | 0.01 | 4.13×10^-58^ |  | -8.54×10^-5^ | 5.09×10^-4^ | 8.67×10^-1^ |  | -1.79×10^-4^ | 1.15×10^-4^ | 1.19×10^-1^ |  | -5.73×10^-5^ | 2.13×10^-4^ | 7.88×10^-1^ |  | 5.66×10^-5^ | 7.21×10^-5^ | 4.33×10^-1^ |  | 2.55×10^-5^ | 5.45×10^-5^ | 6.40×10^-1^ |
| rs1758632 | 9 | C | G | -0.05 | 0.008 | 1.36×10^-09^ |  | 4.05×10^-4^ | 1.27×10^-3^ | 7.49×10^-1^ |  | 6.04×10^-5^ | 8.95×10^-5^ | 5.00×10^-1^ |  | -2.21×10^-4^ | 1.66×10^-4^ | 1.83×10^-1^ |  | -1.03×10^-5^ | 5.62×10^-5^ | 8.55×10^-1^ |  | -4.08×10^-6^ | 4.25×10^-5^ | 9.23×10^-1^ |
| rs17791483 | 9 | G | A | -0.1 | 0.015 | 3.42×10^-12^ |  | -2.54×10^-4^ | 1.22×10^-3^ | 8.35×10^-1^ |  | -1.61×10^-4^ | 1.79×10^-4^ | 3.69×10^-1^ |  | -1.44×10^-5^ | 3.32×10^-4^ | 9.65×10^-1^ |  | -1.46×10^-4^ | 1.12×10^-4^ | 1.95×10^-1^ |  | -1.12×10^-4^ | 8.49×10^-5^ | 1.87×10^-1^ |
| rs2796441 | 9 | A | G | -0.07 | 0.007 | 1.96×10^-22^ |  | 1.24×10^-3^ | 8.55×10^-4^ | 1.48×10^-1^ |  | 1.57×10^-5^ | 8.78×10^-5^ | 8.58×10^-1^ |  | 5.18×10^-5^ | 1.63×10^-4^ | 7.50×10^-1^ |  | -3.15×10^-5^ | 5.52×10^-5^ | 5.67×10^-1^ |  | 7.79×10^-6^ | 4.17×10^-5^ | 8.52×10^-1^ |
| rs10114341 | 9 | C | T | -0.04 | 0.007 | 1.15×10^-08^ |  | 2.01×10^-3^ | 1.49×10^-3^ | 1.78×10^-1^ |  | 2.58×10^-5^ | 8.76×10^-5^ | 7.69×10^-1^ |  | -8.60×10^-5^ | 1.62×10^-4^ | 5.97×10^-1^ |  | 3.40×10^-5^ | 5.50×10^-5^ | 5.37×10^-1^ |  | 7.34×10^-5^ | 4.16×10^-5^ | 7.74×10^-2^ |
| rs687621 | 9 | G | A | 0.043 | 0.008 | 1.35×10^-08^ |  | 5.36×10^-4^ | 1.50×10^-3^ | 7.22×10^-1^ |  | -1.80×10^-4^ | 9.35×10^-5^ | 5.40×10^-2^ |  | 2.20×10^-5^ | 1.73×10^-4^ | 8.99×10^-1^ |  | 6.95×10^-5^ | 5.88×10^-5^ | 2.37×10^-1^ |  | -3.17×10^-5^ | 4.44×10^-5^ | 4.75×10^-1^ |
| rs11257655 | 10 | T | C | 0.074 | 0.009 | 1.97×10^-17^ |  | 7.90×10^-5^ | 1.01×10^-3^ | 9.38×10^-1^ |  | -3.24×10^-5^ | 1.07×10^-4^ | 7.62×10^-1^ |  | 9.28×10^-5^ | 1.98×10^-4^ | 6.40×10^-1^ |  | 3.64×10^-5^ | 6.72×10^-5^ | 5.89×10^-1^ |  | 3.84×10^-5^ | 5.08×10^-5^ | 4.50×10^-1^ |
| rs2616132 | 10 | A | G | 0.046 | 0.008 | 6.58×10^-09^ |  | 2.99×10^-4^ | 1.33×10^-3^ | 8.22×10^-1^ |  | 3.32×10^-5^ | 8.69×10^-5^ | 7.03×10^-1^ |  | -1.39×10^-4^ | 1.61×10^-4^ | 3.89×10^-1^ |  | 4.41×10^-5^ | 5.46×10^-5^ | 4.19×10^-1^ |  | -9.17×10^-6^ | 4.12×10^-5^ | 8.24×10^-1^ |
| rs2633310 | 10 | T | G | -0.04 | 0.008 | 2.38×10^-08^ |  | -1.73×10^-3^ | 1.38×10^-3^ | 2.09×10^-1^ |  | -1.59×10^-4^ | 8.77×10^-5^ | 6.95×10^-2^ |  | -5.52×10^-5^ | 1.63×10^-4^ | 7.34×10^-1^ |  | -4.36×10^-5^ | 5.51×10^-5^ | 4.28×10^-1^ |  | -5.12×10^-5^ | 4.16×10^-5^ | 2.18×10^-1^ |
| rs753270 | 10 | T | C | -0.05 | 0.008 | 2.70×10^-11^ |  | 1.31×10^-3^ | 1.17×10^-3^ | 2.61×10^-1^ |  | -5.88×10^-6^ | 8.85×10^-5^ | 9.47×10^-1^ |  | -1.36×10^-5^ | 1.64×10^-4^ | 9.34×10^-1^ |  | -3.96×10^-5^ | 5.56×10^-5^ | 4.77×10^-1^ |  | 3.20×10^-5^ | 4.20×10^-5^ | 4.46×10^-1^ |
| rs7923866 | 10 | T | C | -0.1 | 0.007 | 9.34×10^-40^ |  | -1.37×10^-3^ | 6.40×10^-4^ | 3.25×10^-2^ |  | -6.30×10^-5^ | 8.94×10^-5^ | 4.81×10^-1^ |  | 1.85×10^-4^ | 1.66×10^-4^ | 2.65×10^-1^ |  | 1.35×10^-5^ | 5.62×10^-5^ | 8.10×10^-1^ |  | -3.52×10^-5^ | 4.24×10^-5^ | 4.07×10^-1^ |
| rs7903146 | 10 | T | C | 0.306 | 0.008 | 1.33×10^-347^ |  | 7.49×10^-5^ | 2.17×10^-4^ | 7.31×10^-1^ |  | -1.09×10^-4^ | 9.56×10^-5^ | 2.53×10^-1^ |  | -3.46×10^-4^ | 1.77×10^-4^ | 5.07×10^-2^ |  | -1.73×10^-5^ | 6.00×10^-5^ | 7.73×10^-1^ |  | 2.29×10^-5^ | 4.54×10^-5^ | 6.14×10^-1^ |
| rs4918796 | 10 | C | T | 0.062 | 0.009 | 4.01×10^-13^ |  | -1.31×10^-3^ | 1.17×10^-3^ | 2.63×10^-1^ |  | -8.31×10^-5^ | 1.05×10^-4^ | 4.28×10^-1^ |  | -4.80×10^-5^ | 1.95×10^-4^ | 8.05×10^-1^ |  | -9.29×10^-5^ | 6.59×10^-5^ | 1.59×10^-1^ |  | -2.73×10^-5^ | 4.98×10^-5^ | 5.83×10^-1^ |
| rs2421016 | 10 | T | C | -0.05 | 0.007 | 1.48×10^-10^ |  | 7.27×10^-4^ | 1.32×10^-3^ | 5.82×10^-1^ |  | -2.98×10^-5^ | 8.70×10^-5^ | 7.32×10^-1^ |  | -2.51×10^-4^ | 1.61×10^-4^ | 1.20×10^-1^ |  | 2.51×10^-5^ | 5.46×10^-5^ | 6.46×10^-1^ |  | 8.36×10^-6^ | 4.13×10^-5^ | 8.40×10^-1^ |
| rs2237892 | 11 | T | C | -0.1 | 0.016 | 8.75×10^-10^ |  | 1.51×10^-3^ | 1.28×10^-3^ | 2.38×10^-1^ |  | 1.01×10^-4^ | 1.77×10^-4^ | 5.69×10^-1^ |  | 6.39×10^-5^ | 3.28×10^-4^ | 8.45×10^-1^ |  | 3.19×10^-4^ | 1.11×10^-4^ | 4.06×10^-3^ |  | -6.90×10^-5^ | 8.39×10^-5^ | 4.11×10^-1^ |
| rs5215 | 11 | C | T | 0.068 | 0.007 | 2.09×10^-20^ |  | 1.37×10^-3^ | 9.29×10^-4^ | 1.39×10^-1^ |  | 1.56×10^-4^ | 9.05×10^-5^ | 8.51×10^-2^ |  | 5.86×10^-5^ | 1.68×10^-4^ | 7.27×10^-1^ |  | -1.38×10^-4^ | 5.69×10^-5^ | 1.54×10^-2^ |  | -6.59×10^-6^ | 4.30×10^-5^ | 8.78×10^-1^ |
| rs7929543 | 11 | C | A | 0.083 | 0.014 | 2.20×10^-09^ |  | 7.22×10^-4^ | 1.32×10^-3^ | 5.83×10^-1^ |  | 2.00×10^-4^ | 1.57×10^-4^ | 2.03×10^-1^ |  | 3.31×10^-4^ | 2.91×10^-4^ | 2.56×10^-1^ |  | 8.47×10^-5^ | 9.87×10^-5^ | 3.91×10^-1^ |  | -6.62×10^-5^ | 7.46×10^-5^ | 3.75×10^-1^ |
| rs1552224 | 11 | C | A | -0.1 | 0.01 | 8.64×10^-25^ |  | 1.30×10^-3^ | 8.05×10^-4^ | 1.07×10^-1^ |  | -1.67×10^-4^ | 1.20×10^-4^ | 1.62×10^-1^ |  | 1.86×10^-4^ | 2.22×10^-4^ | 4.02×10^-1^ |  | -4.40×10^-5^ | 7.51×10^-5^ | 5.58×10^-1^ |  | 8.84×10^-5^ | 5.68×10^-5^ | 1.19×10^-1^ |
| rs10830963 | 11 | G | C | 0.091 | 0.008 | 5.85×10^-30^ |  | 4.66×10^-5^ | 7.44×10^-4^ | 9.50×10^-1^ |  | -1.38×10^-4^ | 9.71×10^-5^ | 1.55×10^-1^ |  | -8.96×10^-5^ | 1.80×10^-4^ | 6.19×10^-1^ |  | -3.90×10^-5^ | 6.10×10^-5^ | 5.22×10^-1^ |  | 7.51×10^-5^ | 4.61×10^-5^ | 1.03×10^-1^ |
| rs7931302 | 11 | C | A | 0.046 | 0.008 | 7.65×10^-09^ |  | 2.62×10^-3^ | 1.47×10^-3^ | 7.35×10^-1^ |  | -1.17×10^-4^ | 9.72×10^-5^ | 2.28×10^-1^ |  | 1.26×10^-4^ | 1.80×10^-4^ | 4.83×10^-1^ |  | -1.33×10^-5^ | 6.10×10^-5^ | 8.28×10^-1^ |  | -1.33×10^-5^ | 4.61×10^-5^ | 7.74×10^-1^ |
| rs67232546 | 11 | T | C | 0.06 | 0.01 | 4.66×10^-10^ |  | -1.66×10^-3^ | 1.25×10^-3^ | 1.83×10^-1^ |  | -1.46×10^-4^ | 1.07×10^-4^ | 1.71×10^-1^ |  | 4.54×10^-5^ | 1.98×10^-4^ | 8.19×10^-1^ |  | 3.81×10^-5^ | 6.72×10^-5^ | 5.71×10^-1^ |  | 6.41×10^-5^ | 5.07×10^-5^ | 2.06×10^-1^ |
| rs12299509 | 12 | G | A | 0.047 | 0.007 | 2.09×10^-10^ |  | 2.51×10^-4^ | 1.30×10^-3^ | 8.47×10^-1^ |  | -1.72×10^-4^ | 8.70×10^-5^ | 4.74×10^-2^ |  | 3.96×10^-5^ | 1.61×10^-4^ | 8.06×10^-1^ |  | 4.23×10^-5^ | 5.46×10^-5^ | 4.39×10^-1^ |  | 5.51×10^-5^ | 4.13×10^-5^ | 1.82×10^-1^ |
| rs11048456 | 12 | C | T | 0.049 | 0.008 | 2.97×10^-09^ |  | 6.25×10^-4^ | 1.44×10^-3^ | 6.65×10^-1^ |  | -6.00×10^-5^ | 1.01×10^-4^ | 5.53×10^-1^ |  | 1.10×10^-5^ | 1.88×10^-4^ | 9.54×10^-1^ |  | -7.96×10^-5^ | 6.37×10^-5^ | 2.11×10^-1^ |  | 6.05×10^-5^ | 4.81×10^-5^ | 2.09×10^-1^ |
| rs10842994 | 12 | T | C | -0.08 | 0.009 | 1.02×10^-16^ |  | 1.60×10^-3^ | 1.01×10^-3^ | 1.11×10^-1^ |  | 3.24×10^-5^ | 1.09×10^-4^ | 7.67×10^-1^ |  | -3.19×10^-5^ | 2.02×10^-4^ | 8.75×10^-1^ |  | -3.84×10^-5^ | 6.85×10^-5^ | 5.75×10^-1^ |  | 5.17×10^-5^ | 5.18×10^-5^ | 3.18×10^-1^ |
| rs2261181 | 12 | T | C | 0.099 | 0.012 | 9.18×10^-17^ |  | 1.18×10^-3^ | 1.05×10^-3^ | 2.60×10^-1^ |  | -1.32×10^-4^ | 1.48×10^-4^ | 3.73×10^-1^ |  | -1.97×10^-4^ | 2.74×10^-4^ | 4.73×10^-1^ |  | 6.72×10^-5^ | 9.30×10^-5^ | 4.70×10^-1^ |  | -2.43×10^-5^ | 7.02×10^-5^ | 7.29×10^-1^ |
| rs1480474 | 12 | G | A | 0.041 | 0.007 | 1.68×10^-08^ |  | -1.64×10^-3^ | 1.50×10^-3^ | 2.75×10^-1^ |  | -6.31×10^-5^ | 8.84×10^-5^ | 4.75×10^-1^ |  | -2.14×10^-4^ | 1.64×10^-4^ | 1.92×10^-1^ |  | -1.91×10^-5^ | 5.55×10^-5^ | 7.30×10^-1^ |  | -7.33×10^-5^ | 4.19×10^-5^ | 8.05×10^-2^ |
| rs7138300 | 12 | C | T | 0.044 | 0.007 | 5.65×10^-10^ |  | 1.02×10^-3^ | 1.38×10^-3^ | 4.58×10^-1^ |  | -1.69×10^-5^ | 8.76×10^-5^ | 8.47×10^-1^ |  | -1.70×10^-5^ | 1.63×10^-4^ | 9.17×10^-1^ |  | -1.08×10^-5^ | 5.50×10^-5^ | 8.44×10^-1^ |  | 5.63×10^-5^ | 4.16×10^-5^ | 1.76×10^-1^ |
| rs11107116 | 12 | T | G | 0.047 | 0.009 | 3.75×10^-08^ |  | 1.14×10^-3^ | 1.55×10^-3^ | 4.62×10^-1^ |  | -1.37×10^-4^ | 1.04×10^-4^ | 1.90×10^-1^ |  | 2.57×10^-4^ | 1.94×10^-4^ | 1.84×10^-1^ |  | -1.58×10^-5^ | 6.56×10^-5^ | 8.09×10^-1^ |  | 1.64×10^-5^ | 4.95×10^-5^ | 7.41×10^-1^ |
| rs61953351 | 12 | T | G | -0.07 | 0.009 | 1.98×10^-14^ |  | 4.94×10^-4^ | 9.97×10^-4^ | 6.20×10^-1^ |  | 2.82×10^-5^ | 1.00×10^-4^ | 7.78×10^-1^ |  | 1.57×10^-4^ | 1.86×10^-4^ | 3.98×10^-1^ |  | -1.32×10^-4^ | 6.30×10^-5^ | 3.66×10^-2^ |  | -4.16×10^-5^ | 4.76×10^-5^ | 3.82×10^-1^ |
| rs940904 | 12 | G | A | -0.05 | 0.008 | 2.08×10^-09^ |  | -1.61×10^-3^ | 1.38×10^-3^ | 2.43×10^-1^ |  | -1.09×10^-5^ | 9.90×10^-5^ | 9.13×10^-1^ |  | 5.26×10^-5^ | 1.84×10^-4^ | 7.75×10^-1^ |  | -2.58×10^-5^ | 6.22×10^-5^ | 6.78×10^-1^ |  | -1.31×10^-5^ | 4.70×10^-5^ | 7.81×10^-1^ |
| rs825476 | 12 | C | T | -0.05 | 0.007 | 6.80×10^-13^ |  | -9.01×10^-4^ | 1.17×10^-3^ | 4.41×10^-1^ |  | -1.48×10^-4^ | 8.79×10^-5^ | 9.23×10^-2^ |  | 2.06×10^-5^ | 1.63×10^-4^ | 9.00×10^-1^ |  | 9.49×10^-5^ | 5.52×10^-5^ | 8.58×10^-2^ |  | -9.80×10^-5^ | 4.17×10^-5^ | 1.88×10^-2^ |
| rs576674 | 13 | G | A | 0.065 | 0.01 | 1.79×10^-11^ |  | -8.21×10^-5^ | 1.24×10^-3^ | 9.47×10^-1^ |  | 5.19×10^-5^ | 1.16×10^-4^ | 6.56×10^-1^ |  | -1.51×10^-4^ | 2.16×10^-4^ | 4.84×10^-1^ |  | 1.67×10^-4^ | 7.31×10^-5^ | 2.23×10^-2^ |  | 5.02×10^-6^ | 5.52×10^-5^ | 9.28×10^-1^ |
| rs963740 | 13 | T | A | -0.05 | 0.009 | 2.23×10^-08^ |  | -5.43×10^-4^ | 1.38×10^-3^ | 6.95×10^-1^ |  | 4.07×10^-5^ | 9.53×10^-5^ | 6.69×10^-1^ |  | 1.40×10^-4^ | 1.77×10^-4^ | 4.28×10^-1^ |  | 1.12×10^-4^ | 5.99×10^-5^ | 6.18×10^-2^ |  | -6.17×10^-5^ | 4.52×10^-5^ | 1.72×10^-1^ |
| rs1359790 | 13 | A | G | -0.08 | 0.008 | 2.80×10^-23^ |  | 1.13×10^-3^ | 8.38×10^-4^ | 1.79×10^-1^ |  | -3.63×10^-5^ | 9.58×10^-5^ | 7.05×10^-1^ |  | 1.17×10^-4^ | 1.78×10^-4^ | 5.09×10^-1^ |  | -2.35×10^-5^ | 6.02×10^-5^ | 6.97×10^-1^ |  | -8.98×10^-6^ | 4.55×10^-5^ | 8.43×10^-1^ |
| rs7144011 | 14 | T | G | 0.048 | 0.009 | 1.64×10^-08^ |  | -1.06×10^-3^ | 1.53×10^-3^ | 4.88×10^-1^ |  | 1.09×10^-4^ | 1.06×10^-4^ | 3.03×10^-1^ |  | -2.20×10^-4^ | 1.96×10^-4^ | 2.61×10^-1^ |  | -7.40×10^-5^ | 6.64×10^-5^ | 2.65×10^-1^ |  | -8.01×10^-6^ | 5.02×10^-5^ | 8.73×10^-1^ |
| rs4502156 | 15 | C | T | -0.04 | 0.007 | 1.66×10^-08^ |  | -9.00×10^-4^ | 1.48×10^-3^ | 5.43×10^-1^ |  | 5.64×10^-5^ | 8.74×10^-5^ | 5.19×10^-1^ |  | 2.00×10^-4^ | 1.62×10^-4^ | 2.16×10^-1^ |  | 4.96×10^-6^ | 5.49×10^-5^ | 9.28×10^-1^ |  | -1.02×10^-4^ | 4.15×10^-5^ | 1.43×10^-2^ |
| rs982077 | 15 | A | G | 0.045 | 0.007 | 2.58×10^-10^ |  | -1.28×10^-3^ | 1.35×10^-3^ | 3.41×10^-1^ |  | 7.86×10^-5^ | 8.78×10^-5^ | 3.70×10^-1^ |  | 2.13×10^-4^ | 1.63×10^-4^ | 1.91×10^-1^ |  | -4.85×10^-5^ | 5.51×10^-5^ | 3.79×10^-1^ |  | -2.42×10^-5^ | 4.17×10^-5^ | 5.62×10^-1^ |
| rs7177055 | 15 | G | A | -0.06 | 0.008 | 2.75×10^-16^ |  | -7.33×10^-4^ | 1.04×10^-3^ | 4.81×10^-1^ |  | 1.82×10^-4^ | 9.65×10^-5^ | 5.94×10^-2^ |  | 3.70×10^-5^ | 1.79×10^-4^ | 8.36×10^-1^ |  | 2.69×10^-5^ | 6.06×10^-5^ | 6.57×10^-1^ |  | -1.35×10^-5^ | 4.58×10^-5^ | 7.68×10^-1^ |
| rs4932143 | 15 | G | C | 0.057 | 0.009 | 5.51×10^-11^ |  | -2.39×10^-4^ | 1.18×10^-3^ | 8.40×10^-1^ |  | 5.81×10^-5^ | 9.67×10^-5^ | 5.48×10^-1^ |  | 3.17×10^-4^ | 1.79×10^-4^ | 7.75×10^-2^ |  | 3.33×10^-5^ | 6.08×10^-5^ | 5.84×10^-1^ |  | 3.15×10^-5^ | 4.59×10^-5^ | 4.92×10^-1^ |
| rs12910825 | 15 | G | A | 0.052 | 0.007 | 2.16×10^-12^ |  | 2.94×10^-4^ | 1.22×10^-3^ | 8.10×10^-1^ |  | -9.34×10^-5^ | 9.06×10^-5^ | 3.03×10^-1^ |  | 1.33×10^-4^ | 1.68×10^-4^ | 4.30×10^-1^ |  | 2.93×10^-5^ | 5.69×10^-5^ | 6.07×10^-1^ |  | 2.48×10^-5^ | 4.30×10^-5^ | 5.64×10^-1^ |
| rs9940149 | 16 | A | G | -0.06 | 0.01 | 9.29×10^-10^ |  | -1.03×10^-6^ | 1.36×10^-3^ | 4.50×10^-1^ |  | -2.18×10^-4^ | 1.14×10^-4^ | 5.50×10^-2^ |  | 9.48×10^-5^ | 2.11×10^-4^ | 6.53×10^-1^ |  | 7.55×10^-5^ | 7.13×10^-5^ | 2.90×10^-1^ |  | 2.86×10^-5^ | 5.39×10^-5^ | 5.96×10^-1^ |
| rs7185735 | 16 | G | A | 0.106 | 0.007 | 1.59×10^-47^ |  | 1.27×10^-5^ | 5.86×10^-4^ | 9.83×10^-1^ |  | 2.37×10^-5^ | 8.89×10^-5^ | 7.90×10^-1^ |  | -1.70×10^-4^ | 1.65×10^-4^ | 3.02×10^-1^ |  | -4.27×10^-6^ | 5.59×10^-5^ | 9.39×10^-1^ |  | 4.37×10^-5^ | 4.22×10^-5^ | 3.00×10^-1^ |
| rs244415 | 16 | A | G | -0.05 | 0.008 | 3.88×10^-09^ |  | -4.18×10^-4^ | 1.32×10^-3^ | 7.52×10^-1^ |  | -2.70×10^-5^ | 8.85×10^-5^ | 7.60×10^-1^ |  | -1.07×10^-4^ | 1.64×10^-4^ | 5.16×10^-1^ |  | -3.35×10^-5^ | 5.56×10^-5^ | 5.46×10^-1^ |  | 2.45×10^-5^ | 4.20×10^-5^ | 5.59×10^-1^ |
| rs77258096 | 16 | A | C | -0.12 | 0.013 | 1.78×10^-18^ |  | 3.44×10^-4^ | 8.63×10^-4^ | 6.90×10^-1^ |  | -7.54×10^-5^ | 1.45×10^-4^ | 6.02×10^-1^ |  | -7.25×10^-5^ | 2.68×10^-4^ | 7.87×10^-1^ |  | 7.93×10^-5^ | 9.09×10^-5^ | 3.82×10^-1^ |  | 1.57×10^-4^ | 6.86×10^-5^ | 2.18×10^-2^ |
| rs2925979 | 16 | T | C | 0.053 | 0.008 | 9.06×10^-12^ |  | 1.81×10^-4^ | 1.23×10^-3^ | 8.83×10^-1^ |  | -6.33×10^-5^ | 9.47×10^-5^ | 5.04×10^-1^ |  | -1.53×10^-5^ | 1.76×10^-4^ | 9.31×10^-1^ |  | -1.14×10^-4^ | 5.95×10^-5^ | 5.55×10^-2^ |  | 9.73×10^-6^ | 4.49×10^-5^ | 8.29×10^-1^ |
| rs8068804 | 17 | A | G | 0.059 | 0.008 | 4.41×10^-14^ |  | -1.96×10^-3^ | 1.10×10^-3^ | 7.41×10^-2^ |  | 1.42×10^-4^ | 9.26×10^-5^ | 1.26×10^-1^ |  | 1.09×10^-4^ | 1.72×10^-4^ | 5.27×10^-1^ |  | -1.22×10^-4^ | 5.82×10^-5^ | 3.61×10^-2^ |  | 3.19×10^-5^ | 4.39×10^-5^ | 4.68×10^-1^ |
| rs12945601 | 17 | T | C | 0.048 | 0.008 | 1.72×10^-09^ |  | 3.60×10^-5^ | 1.30×10^-3^ | 9.78×10^-1^ |  | -3.50×10^-5^ | 8.98×10^-5^ | 6.96×10^-1^ |  | 3.52×10^-5^ | 1.67×10^-4^ | 8.33×10^-1^ |  | -3.59×10^-5^ | 5.64×10^-5^ | 5.25×10^-1^ |  | 4.49×10^-5^ | 4.26×10^-5^ | 2.92×10^-1^ |
| rs11651755 | 17 | C | T | 0.074 | 0.008 | 8.98×10^-22^ |  | -4.94×10^-4^ | 8.19×10^-4^ | 5.47×10^-1^ |  | -3.94×10^-5^ | 8.73×10^-5^ | 6.51×10^-1^ |  | 5.99×10^-5^ | 1.62×10^-4^ | 7.11×10^-1^ |  | 8.88×10^-6^ | 5.48×10^-5^ | 8.71×10^-1^ |  | -3.13×10^-5^ | 4.14×10^-5^ | 4.50×10^-1^ |
| rs17405722 | 17 | A | G | 0.087 | 0.015 | 2.28×10^-09^ |  | -6.69×10^-4^ | 1.31×10^-3^ | 6.10×10^-1^ |  | -8.53×10^-5^ | 1.64×10^-4^ | 6.04×10^-1^ |  | -2.27×10^-4^ | 3.05×10^-4^ | 4.56×10^-1^ |  | -1.34×10^-4^ | 1.03×10^-4^ | 1.95×10^-1^ |  | -8.45×10^-5^ | 7.80×10^-5^ | 2.79×10^-1^ |
| rs9911983 | 17 | C | T | -0.04 | 0.007 | 4.82×10^-08^ |  | 2.87×10^-4^ | 1.54×10^-3^ | 8.52×10^-1^ |  | 5.40×10^-5^ | 8.77×10^-5^ | 5.38×10^-1^ |  | 1.18×10^-4^ | 1.63×10^-4^ | 4.70×10^-1^ |  | 2.84×10^-5^ | 5.51×10^-5^ | 6.06×10^-1^ |  | -1.13×10^-5^ | 4.16×10^-5^ | 7.86×10^-1^ |
| rs9894220 | 17 | G | A | -0.06 | 0.008 | 1.52×10^-13^ |  | 9.91×10^-4^ | 1.04×10^-3^ | 3.42×10^-1^ |  | 4.92×10^-5^ | 8.76×10^-5^ | 5.75×10^-1^ |  | -2.20×10^-5^ | 1.62×10^-4^ | 8.92×10^-1^ |  | 2.56×10^-5^ | 5.50×10^-5^ | 6.42×10^-1^ |  | 1.33×10^-5^ | 4.16×10^-5^ | 7.49×10^-1^ |
| rs302864 | 17 | A | G | 0.071 | 0.013 | 2.46×10^-08^ |  | 1.30×10^-3^ | 1.51×10^-3^ | 3.90×10^-1^ |  | -9.44×10^-5^ | 1.54×10^-4^ | 5.40×10^-1^ |  | 2.34×10^-4^ | 2.85×10^-4^ | 4.12×10^-1^ |  | 2.84×10^-5^ | 9.66×10^-5^ | 7.69×10^-1^ |  | 9.33×10^-5^ | 7.30×10^-5^ | 2.01×10^-1^ |
| rs17631783 | 17 | T | C | -0.05 | 0.009 | 3.95×10^-08^ |  | -2.16×10^-3^ | 1.41×10^-3^ | 1.28×10^-1^ |  | 1.26×10^-4^ | 9.90×10^-5^ | 2.02×10^-1^ |  | 4.26×10^-4^ | 1.84×10^-4^ | 2.05×10^-2^ |  | 7.38×10^-5^ | 6.22×10^-5^ | 2.36×10^-1^ |  | 7.73×10^-6^ | 4.70×10^-5^ | 8.69×10^-1^ |
| rs7240767 | 18 | C | T | 0.045 | 0.008 | 2.16×10^-08^ |  | -5.28×10^-4^ | 1.38×10^-3^ | 7.02×10^-1^ |  | 2.01×10^-5^ | 8.93×10^-5^ | 8.22×10^-1^ |  | 2.97×10^-4^ | 1.66×10^-4^ | 7.24×10^-2^ |  | 8.01×10^-6^ | 5.61×10^-5^ | 8.86×10^-1^ |  | 1.12×10^-4^ | 4.24×10^-5^ | 8.17×10^-3^ |
| rs12970134 | 18 | A | G | 0.056 | 0.008 | 5.31×10^-12^ |  | 1.03×10^-3^ | 1.23×10^-3^ | 4.03×10^-1^ |  | -1.83×10^-4^ | 9.81×10^-5^ | 6.22×10^-2^ |  | -2.10×10^-4^ | 1.82×10^-4^ | 2.49×10^-1^ |  | -6.03×10^-5^ | 6.16×10^-5^ | 3.28×10^-1^ |  | -3.56×10^-5^ | 4.66×10^-5^ | 4.44×10^-1^ |
| rs10401969 | 19 | C | T | 0.092 | 0.013 | 4.13×10^-12^ |  | -2.06×10^-4^ | 1.24×10^-3^ | 8.68×10^-1^ |  | 1.29×10^-4^ | 1.63×10^-4^ | 4.29×10^-1^ |  | -5.43×10^-4^ | 3.03×10^-4^ | 7.33×10^-2^ |  | 6.33×10^-5^ | 1.03×10^-4^ | 5.38×10^-1^ |  | 4.71×10^-5^ | 7.76×10^-5^ | 5.43×10^-1^ |
| rs8108269 | 19 | G | T | 0.064 | 0.008 | 3.11×10^-16^ |  | 1.04×10^-3^ | 1.05×10^-3^ | 3.21×10^-1^ |  | 6.69×10^-5^ | 9.68×10^-5^ | 4.90×10^-1^ |  | -1.41×10^-4^ | 1.79×10^-4^ | 4.31×10^-1^ |  | -4.96×10^-5^ | 6.08×10^-5^ | 4.14×10^-1^ |  | -2.35×10^-5^ | 4.59×10^-5^ | 6.09×10^-1^ |
| rs6515236 | 20 | C | A | -0.05 | 0.009 | 3.34×10^-08^ |  | -3.29×10^-4^ | 1.39×10^-3^ | 8.13×10^-1^ |  | 9.77×10^-6^ | 1.01×10^-4^ | 9.23×10^-1^ |  | 2.59×10^-4^ | 1.87×10^-4^ | 1.65×10^-1^ |  | 3.52×10^-5^ | 6.33×10^-5^ | 5.78×10^-1^ |  | -2.12×10^-5^ | 4.78×10^-5^ | 6.58×10^-1^ |
| rs6059662 | 20 | A | G | -0.04 | 0.008 | 1.51×10^-08^ |  | 8.21×10^-4^ | 1.43×10^-3^ | 5.67×10^-1^ |  | -7.10×10^-5^ | 9.19×10^-5^ | 4.40×10^-1^ |  | 9.00×10^-5^ | 1.70×10^-4^ | 5.97×10^-1^ |  | -8.54×10^-6^ | 5.77×10^-5^ | 8.82×10^-1^ |  | 1.67×10^-5^ | 4.36×10^-5^ | 7.02×10^-1^ |
| rs4810426 | 20 | T | C | 0.073 | 0.013 | 2.15×10^-08^ |  | 2.05×10^-3^ | 1.42×10^-3^ | 1.48×10^-1^ |  | 6.94×10^-5^ | 1.49×10^-4^ | 6.41×10^-1^ |  | 1.20×10^-4^ | 2.76×10^-4^ | 6.62×10^-1^ |  | 2.46×10^-5^ | 9.34×10^-5^ | 7.93×10^-1^ |  | 1.65×10^-5^ | 7.06×10^-5^ | 8.15×10^-1^ |
| rs6066138 | 20 | A | G | -0.05 | 0.008 | 1.93×10^-09^ |  | -3.20×10^-3^ | 1.37×10^-3^ | 1.93×10^-2^ |  | 3.31×10^-6^ | 9.65×10^-5^ | 9.73×10^-1^ |  | 2.19×10^-4^ | 1.79×10^-4^ | 2.22×10^-1^ |  | 2.20×10^-5^ | 6.06×10^-5^ | 7.17×10^-1^ |  | 6.34×10^-5^ | 4.58×10^-5^ | 1.66×10^-1^ |
| rs16988333 | 22 | G | A | -0.07 | 0.013 | 9.17×10^-09^ |  | 6.71×10^-4^ | 1.40×10^-3^ | 6.31×10^-1^ |  | 1.31×10^-4^ | 1.49×10^-4^ | 3.79×10^-1^ |  | 2.75×10^-4^ | 2.77×10^-4^ | 3.20×10^-1^ |  | 4.36×10^-5^ | 9.38×10^-5^ | 6.42×10^-1^ |  | 7.16×10^-5^ | 7.08×10^-5^ | 3.12×10^-1^ |
| rs4823182 | 22 | G | A | 0.048 | 0.008 | 3.36×10^-10^ |  | -6.14×10^-4^ | 1.33×10^-3^ | 6.45×10^-1^ |  | -1.70×10^-5^ | 9.23×10^-5^ | 8.54×10^-1^ |  | -2.65×10^-4^ | 1.71×10^-4^ | 1.21×10^-1^ |  | -1.13×10^-5^ | 5.80×10^-5^ | 8.46×10^-1^ |  | 3.29×10^-5^ | 4.38×10^-5^ | 4.52×10^-1^ |

T2DM, type 2 diabetes mellitus; SNP, single-nucleotide polymorphism; Chr, chromosome; EA, effect allele; NEA, non-effect allele; SE, standard error; SSTI: skin and soft tissue infections; UTI: urinary tract infections; GUI: genito-urinary infection.
